# The use of traditional Chinese medicines in relieving exercise-induced fatigue

**DOI:** 10.3389/fphar.2022.969827

**Published:** 2022-07-22

**Authors:** Yuzhou Liu, Congying Li, Xiaofei Shen, Yue Liu

**Affiliations:** ^1^ School of Leisure Sports, Chengdu Sport University, Chengdu, China; ^2^ State Key Laboratory of Southwestern Chinese Medicine Resources, School of Ethnic Medicine, Chengdu University of Traditional Chinese Medicine, Chengdu, China; ^3^ TCM Regulating Metabolic Diseases Key Laboratory of Sichuan Province, Hospital of Chengdu University of Traditional Chinese Medicine, Chengdu University of Traditional Chinese Medicine, Chengdu, China

**Keywords:** exercise-induced fatigue, traditional Chinese medicines, relieving, molecular mechanism, medicinal and edible values

## Abstract

Exercise-induced fatigue is a non-pathological fatigue and indicated by a reduction of muscle performance that is caused by excessive physical activity. It seriously affects the daily lives of people, in particular athletes, military personnel, and manual laborers. In recent years, increasing attention has been paid to improving the adverse effect of exercise-induced fatigue on people’s daily activities. Thus, studies and applications of traditional Chinese medicines (TCMs) in relieving exercise-induced fatigue have become the focus because of their good curative effects with fewer side effects. This review aims to document and summarize the critical and comprehensive information about the biological processes of exercise-induced fatigue, and to know the types of TCMs, their active components, and possible molecular mechanisms in alleviating exercise-induced fatigue. The peripheral and central mechanisms that cause exercise-induced fatigue have been summarized. A total of 47 exercise-induced fatigue relief TCMs have been collected, mostly including the types of visceral function regulation and emotional adjustment TCMs. Polysaccharides, terpenes, flavonoids/polyphenols are demonstrated to be the major bioactive components. The underlying molecular mechanisms are mainly related to the improvement of energy metabolism, elimination of excess metabolites, inhibition of oxidative stress and inflammatory response, regulation of HPA axis and neurotransmitters. Although current results are obtained mostly from animal models, the clinic trials are still insufficient, and a very few TCMs have been reported to possess potential hepatotoxicity. These findings still offer great reference value, and the significant efficacy in relieving exercise-induced fatigue is impossible to ignore. This review is expected to give insights into the research and development of new TCMs-derived drugs and health care products in relieving exercise-induced fatigue.

## Graphical Abstract

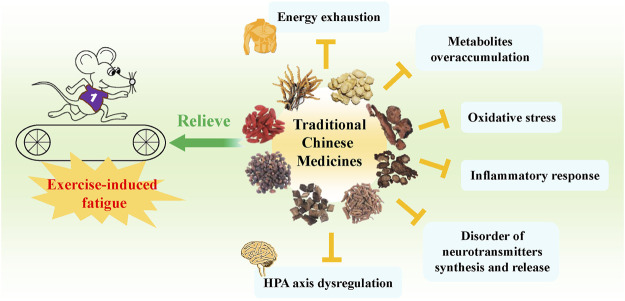

## 1 Introduction

Fatigue is a complex physiological and pathological phenomenon with feelings of exhaustion, tiredness, and weariness or lack of energy as symptoms (Hsiao et al., 2018). A commonly accepted definition of fatigue is that given by Chaudhuri and Behan who defined fatigue as difficulty in initiation of or sustaining voluntary activities, and can be categorized as peripheral and central fatigue (Chaudhuri and Behan, 2004). Fatigue is usually caused by high-intensity physical work and continuous exercise, long-term heavy mental work, hence, leading to body disorders and various diseases, such as aging, depression, cancer, multiple sclerosis, and Parkinson’s disease (Hsiao et al., 2018; Klosterhoff et al., 2018).

Exercise-induced fatigue belongs to non-pathological fatigue, which is a reduction in maximal voluntary muscle force that comes from intense and prolonged physical activities, consequently declining physical performance (Gandevia, 2001; Zhao et al., 2012; Li et al., 2019). The two major theories of exercise-induced fatigue are energy exhaustion and metabolite accumulation. Besides, oxidative stress, inflammation and protective inhibition, as well as functional changes in sympathetic adrenomedullary (SA) system and hypothalamic-pituitary-adrenal (HPA) axis are also demonstrated to be the key contributors to the pathogenesis of exercise-induced fatigue (Chen et al., 2021). Furthermore, severe stress on multiple organs, tissues, and cells is caused by prolonged and high-intensity exercise thus leading to a reduction of work efficiency and exercise performance, which seriously affects the normal production and lives of people, in particular pilots, military personnel, fire-fighters, and athletes. In addition, exercise-induced fatigue requires to be treated with drugs or other interventional strategies because it is not easy to alleviate (Twomey et al., 2017). Therefore, finding exercise-induced fatigue relief drugs with definite efficacy and fewer side effects is extremely necessary.

In recent years, numerous studies demonstrated the significance of traditional Chinese medicines (TCMs) in delaying and relieving exercise-induced fatigue by promoting the ability of antioxidation, enhancing free radical scavenging activity, boosting the immune system, regulating and improving the metabolic balance, etc. (Chen et al., 2010; Luo et al., 2019; Zhou and Jiang, 2019; Tao, 2021). This work summarizes the present understanding of the mechanisms underlying exercise-induced fatigue, introduces a series of exercise-induced fatigue relief TCMs that categorized by their efficacies based on TCM theory. The representative active components and possible molecular mechanisms of TCMs in relieving exercise-induced fatigue are also discussed, thus laying the foundation for further research and providing scientific evidence expected for the development of potential drugs and health care products for relieving exercise-induced fatigue.

## 2 Mechanisms of exercise-induced fatigue

Exercise-induced fatigue mechanisms are not fully understood, however, the peripheral and central mechanisms, including the two major theories and other key pathogenesis of exercise-induced fatigue are generally accepted as the main causes of exercise-induced fatigue.

### 2.1 Peripheral mechanism of exercise-induced fatigue

#### 2.1.1 Energy exhaustion theory

Energy supply is essential for muscle contraction. The main sources of energy for muscle fibers are exhausted during the prolonged and intense exercise, including adenosine triphosphate (ATP), glycogen, and fats within the body system, thus leading to a lack of energy and aerobic capacity of skeletal muscle to complete the required muscle contraction or performed workload (Enoka and Duchateau, 2008; Ament and Verkerke, 2009). The energy materials are consumed differently based on different exercise conditions: The content of ATP and phosphocreatine (PCr) in skeletal muscle decreased during short-time high-intensity exercise, hence, directly resulting to exercise-induced fatigue; Carbohydrates are the primary consumed substrate during moderate exercise; Fats are the mainly consumed energy materials during long endurance exercise (Gastin, 2001; Shulman and Rothman, 2001; Skare et al., 2001). Exercise-induced fatigue also happens with the continuous energy consumption supplied from the ATP and the decrease of the phosphorylation of adenosine diphosphate (ADP) to ATP (Feng, 2003).

#### 2.1.2 Metabolite accumulation theory

Metabolite accumulation theory holds that excess metabolites (such as lactate and amines, etc.) generated from the intense exercise and accumulated in skeletal muscle and blood, hence, resulting in fatigue. Lactate accumulation has been considered one of the most important causes of skeletal muscle fatigue (Westerblad et al., 2002). Long and high-intensity exercise may lead to the dysfunction of ATP production and utilization rates, which cause an increased ATP consumption accompanied by the accumulation of metabolic by-products, such as H^+^ and inorganic phosphate (Pi) (Wang et al., 2021b). The increased concentration of H^+^ (decreased pH) results in a glycolysis inhibition and ATP supply obstruction. The H^+^ accumulation also inhibits Ca^2+^ binding to troponin (Tn), consequently affect cross-bridge cycling and sarcoplasmic reticulum Ca^2+^ pumping, and ultimately contributes to muscle fatigue (Dutka and Lamb, 2000; Westerblad et al., 2002; Bandschapp et al., 2012; Xu et al., 2017). In addition, the concentration of ammonia raised in skeletal muscle during intense exercise. The increased ammonia activates phosphofructokinase and suppresses the oxidation of pyruvate to acetyl CoA, then promotes the production of blood and muscle lactic acids (BLA and MLA), blood urea nitrogen (BUN), creatine kinase (CK), and malondialdehyde (MDA) hence breaking homeostasis and causing fatigue (Takeda et al., 2011; Gough et al., 2021).

#### 2.1.3 Oxidative stress

It has been demonstrated that excessive muscular exercise increases the production of reactive oxygen species (ROS) in body tissues and organs, including myocardial tissue, liver, skeletal muscles, and blood, thus causing an imbalance in the oxidation-antioxidant homeostasis in cells (Powers et al., 2020; Wang et al., 2021a). Low-to-moderate ROS levels are considered to be beneficial to the person’s physical performance, whereas high levels of ROS damage the membrane structure of cells or organelles by attacking biomacromolecules (lipids, proteins, and nucleic acids) (Vasilaki et al., 2017; Lu et al., 2021). In addition, the excessive exercise-induced ROS reduce the activity of skeletal sarcoplasmic reticulum calcium adenosine triphosphatase (Ca^2+^-ATPase), leading to the accumulation of Ca^2+^ in the cytoplasm, and influencing the excitation-contraction coupling of muscle fibers (Uyama et al., 1993). Consequently, the capacity for muscle contraction is reduced, which results in fatigue. Moreover, the elevated endogenous and exogenous ROS destroys mitochondrial functions and inhibits aerobic metabolism which causes exercise-induced fatigue (Cheng et al., 2016).

#### 2.1.4 Inflammation

The prolonged and intense exercise triggers ROS production and oxidative stress and also causes acute and chronic inflammation, consequently leading to a drop in physical performance (Powers et al., 2011). After excessive exercise, pro-inflammatory cytokines such as interleukin-1β (IL-1β), interleukin-6 (IL-6), and tumor necrosis factor-α (TNF-α) levels are increased (Liu et al., 2017; Medrado de Barcellos et al., 2021). It has also been reported that the elevated pro-inflammatory cytokines can activate the nuclear factor kappa-B (NF-κB) and generate a vicious circle of inflammatory response and mitochondrial dysfunction (López-Armada et al., 2013). The impaired mitochondria produce a greater amount of ROS, consequently, resulting in muscle strength decline and fatigue (Vargas and Marino, 2014).

### 2.2 Central mechanism of exercise-induced fatigue

#### 2.2.1 Protective inhibition theory

Central inhibition plays an important role during exercise-induced fatigue and is considered a neurotransmitter mediated defense action (Tanaka et al., 2013). The nerve excitation from the skeletal muscle contraction constantly stimulates the corresponding neurons in the cerebral cortex and maintains excitement during exercise, thus, leading to a continuing consumption of ATP, fatty acids, PCr, and glucose (Hirvonen et al., 1992; Hargreaves, 2015). Subsequently, the cerebral cortex and central nervous system (CNS) switch from excitation to inhibition through the negative feedback regulation mechanism to prevent excessive consumption of energy materials, thus causing fatigue (Qiao et al., 2014).

#### 2.2.2 Neurotransmitter mediated exercise-induced fatigue

The much-studied brain neurotransmitters, such as serotonin (5-HT) and dopamine (DA) were demonstrated to be dominant in accelerating fatigue during intense exercise (Roelands and Meeusen, 2010). 5-HT is a neurotransmitter synthesized from tryptophan (TRP), which can be transported through the blood-brain barrier with the aid of a specific carrier (Roelands and Meeusen, 2010). The concentration of the TRP in both plasma and brain raised, subsequently leads to an increase of 5-HT level in the brain during prolonged exercise (Hu Y. et al., 2015). It has been reported that the high concentration of 5-HT promotes lethargy and perceived exertion, thus inducing the exercise performance restriction and central fatigue (Meeusen et al., 2006). Hyperthermia is a critical limiting factor in long-term exercise, and it has been shown that DA affects core temperature regulation during exercise, which is recognized as being an important fatigue-related neurotransmitter (Zheng and Hasegawa, 2016; Wang et al., 2021b). In addition, an increase in serotonergic activity and a decrease in dopaminergic activity are also demonstrated to be responsible for the exercise-induced fatigue (Meeusen et al., 2007; Leite et al., 2010).

#### 2.2.3 Endocrine mediated exercise-induced fatigue

The endocrine disorder is another aspect of exercise-induced fatigue. It has been reported that the stress response is triggered by intense exercise, thus, activating the SA and HPA axes (Ulrich-Lai and Herman, 2009; Clark and Mach, 2016). Catecholamines (such as norepinephrine and epinephrine) and glucocorticoids were released into the circulation system, hence, resulting in an increased heart rate and blood pressure during the short-term moderate exercise. It has been reported that the key role of catecholamines is to regulate oxidative metabolism, lipoprotein metabolism, glycogen breakdown, and energy expenditure. Therefore, the concentration of catecholamines is positively correlated with the exercise ability during the short term. However, the increased exercise intensity and duration further leads to the decrease of energy substrate, the insufficiency of catecholamine receptors, and the weakening of receptor-mediated signals which result in the failure to enhance exercise ability through compensatory mechanism, although the concentration of catecholamines is still at a high level. Thus, the elevated level of catecholamines cannot enhance the long-term exercise ability, or can even weaken the exercise capacity (Zouhal et al., 2008). In addition, the exercise stress initially enhances the activation of the HPA and increases the cortisol concentration to regulate the energetic, metabolic, and immunologic processes (Grandys et al., 2016). The prolonged and high-intensity exercise initiates a continuous increase of cortisol which inhibits the HPA axis and decreases the level of serum testosterone, thus leading to a decline in physical performance.

## 3 An understanding of exercise-induced fatigue in traditional Chinese medicine

According to TCM theory, exercise-induced fatigue is mainly caused by excessive physical exertion, subsequently causing consumptions of essence (Jing), vital energy (Qi), and spirit (Shen), which led to the imbalance of Yin and Yang, and Qi and blood. The pathogenesis is related to Yin-Yang, Qi-blood deficiency in visceral functions, Qi stagnation, and endogenous toxin, stasis and phlegm. Combined with modern research, the accumulated metabolites during intense exercise, including lactate and H^+^, Pi, and BUN, and free radicals are considered as toxin, stasis and phlegm, which are pathological products based on TCM theory. These accumulated metabolites are also claimed to block meridians and blood vessels, and aggravate fatigue symptoms (Liang and Wen, 2016; Chu et al., 2020; Yang et al., 2022). Therefore, exercise-induced fatigue was classified into three major types by symptoms in TCM, including the physical (accompanied by muscle and bone soreness), visceral (accompanied by spleen and stomach dysfunction, kidney Qi deficiency, etc.), and mental (accompanied by sports insomnia, depression, etc.) (Zhang et al., 2003; Chu et al., 2020). Based on the syndrome differentiation and treatment system, TCM emphasizes the combination of regulation and supplementation by strengthening the body’s resistance into eliminating pathogenic factors by restoring the visceral function and regulating spirit and emotion. Therefore, visceral function regulating TCMs (including Yin nourishing and Yang supporting TCMs, Qi promoting and blood circulating TCMs, and internal heat clearing TCMs), and spirit and emotion adjusting TCMs are most commonly used in clinical practices in the prevention and treatment of exercise-induced fatigue.

## 4 Traditional Chinese medicines in relieving exercise-induced fatigue

### 4.1 Visceral function regulating TCMs in relieving exercise-induced fatigue

**FIGURE 1:**
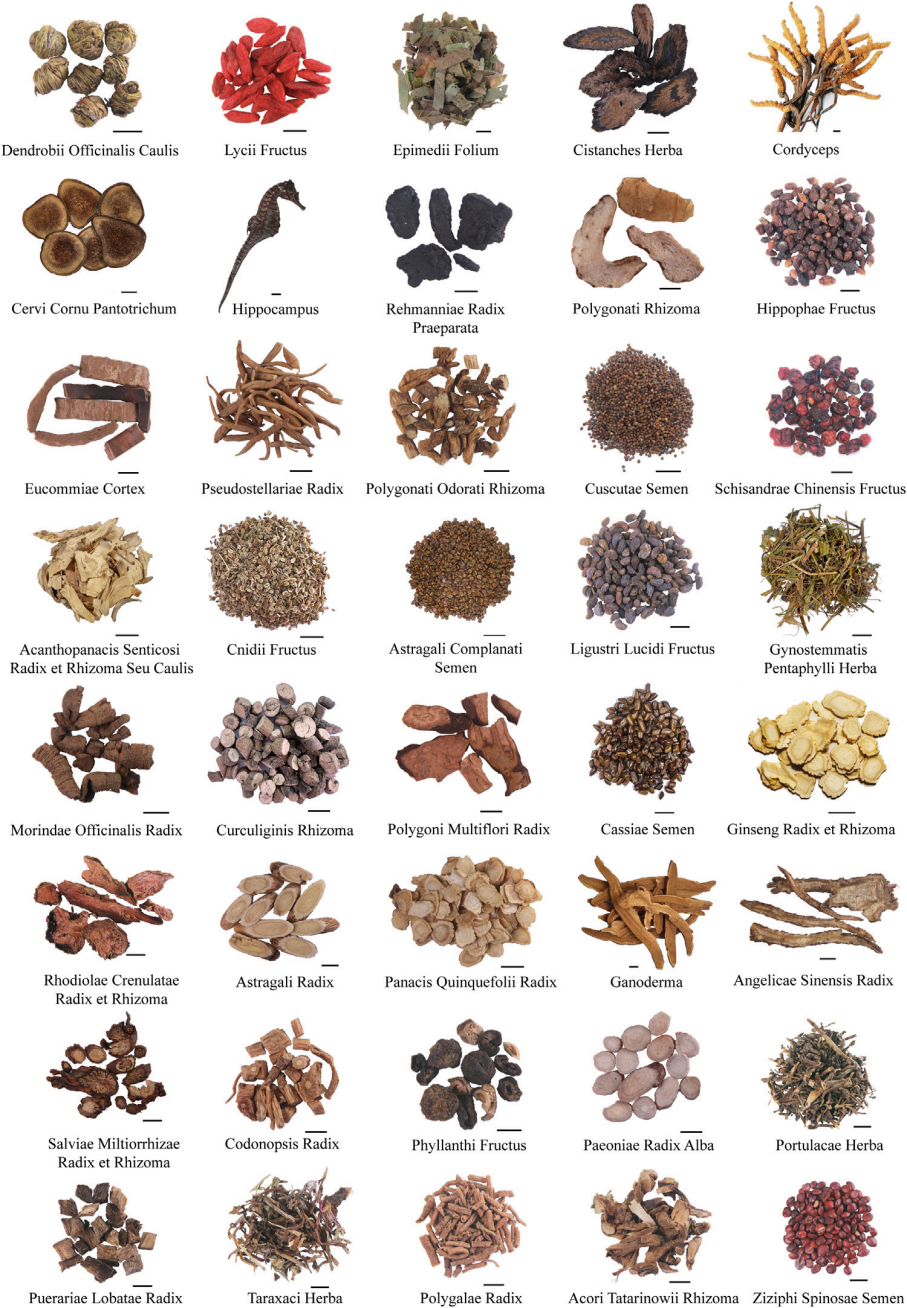
Representative TCMs with relieving effect on exercise-induced fatigue (Scale bar 1 cm).

The body’s metabolism is accelerated during the exercise, resulting in increased energy and materials consumption, metabolites accumulation, blood pH value decrease, and endocrine disorders (Liang and Wen, 2016). TCM theory believes that exercise-induced fatigue is closely related to the visceral function, and visceral function regulating TCMs are commonly used. The frequently used TCMs were illustrated in the following subsections and the representative TCMs are shown in Figure 1.

#### 4.1.1 Yin nourishing and Yang supporting TCMs in relieving exercise-induced fatigue

Dendrobii Officinalis Caulis originated from the fresh or dried stems of *Dendrobium officinale* Kimura et Migo (Fam. Orchidaceae), which has been used as a precious TCM (Chinese Pharmacopoeia Commission, 2020). It is one of the well-accepted tonic medicines in China, and has also been broadly taken as dietary supplements to nourish the stomach, enhance body fluid production, tonify Yin, and clear heat of the internal organs (Zeng et al., 2018; Cao et al., 2020). *D. officinale*’s major bioactive components polysaccharide and flavonoids exhibited strong effects in improving exercise endurance, which is associated in the reduction of metabolite accumulation, increased activities of antioxidant enzymes, and gene and protein expression of peroxisome proliferator-activated receptor-gamma coactivator-1alpha (PGC-1α), nuclear factor-erythroid 2-related factor-2 (Nrf2), and superoxide dismutase 2 (SOD2) (Wei et al., 2017; Wang, 2021a). Studies also indicated that the *D. officinale* polysaccharide and flavonoids could enhance the cell viability of T/B lymphocytes, improve the immune system function, and regulate liver autophagy hence recovering from physical fatigue (Jiang, 2019; Wang et al., 2022a). The ethanol extract of *D. officinale* could improve fatigue resistance in exhausted swimming mice by protecting it against oxidative stress and enhancing the PGC-1α expression (Kim et al., 2020). In addition, the aqueous extract of *D. officinale* could effectively improve the endurance capability of mice against physical fatigue by regulating energy metabolism and nourishing muscle (Tang et al., 2014).

Lycii Fructus is the dried fruit of *Lycium barbarum* L. (Fam. Solanaceae), which has been used as traditional herbal medicine and food supplement for its restorative efficacy and benefiting to the liver and kidney, replenishing vital essence, and improving eyesight (Sze et al., 2008; Gao et al., 2017; Chinese Pharmacopoeia Commission, 2020). According to pharmacological research, *L. barbarum* polysaccharide (LBP) plays an important role as an antioxidant and anti-fatigue agent. It has been illustrated that LBP could enhance the exercise capacity by rising the activities of antioxidant enzymes, reducing free radicals and lipid peroxides, improving BLA tolerance of the skeletal muscle (Zhao et al., 2013; Hu X. Y. et al., 2015; Zhang Y. L. et al., 2015; Wei et al., 2020). Moreover, studies also showed that LBP could attenuate kidney injury by inactivating the NF-κB pathway, activating the Kelch-like ECH-associated protein 1-nuclear factor erythroid 2-related factor 2 (Keap1-NRF2) signaling, and reducing the release of pro-inflammatory cytokines (Wu et al., 2020). Liu et al., synthesized an LBP-SeNPs (selenium nanoparticles) product and demonstrated its activity in beating exercise-induced fatigue by up-regulating glycogen storage and antioxidant enzyme levels, as well as metabolic modulation activity (Liu et al., 2021). Furthermore, *L. barbarum* leaves showed similar anti-fatigue effects (Yue, 2016).

Epimedii Folium contains the dried leaves of *Epimedium brevicornu* Maxim., *E. sagittatum* (Sieb. et Zucc.) Maxim., *E. pubescens* Maxim., *E. koreanum* Nakai and relative plants in the same genus (Fam. Berberidaceae) with pharmacological effects in reinforcing the kidney Yang, strengthening the tendons and bones, and dispelling wind dampness (Chen et al., 2015; Chinese Pharmacopoeia Commission, 2020). Both of the aqueous and ethanol extracts of *E. brevicornu* could improve the endurance capability of the exercise-induced animal models by lowering the serum levels of BUN, BLA, and increasing the content of liver glycogen (Li et al., 2021b). The aqueous extract of *E. davidii* could increase the glycogen storage of the liver and muscle, and rise the serum testosterone level and hemoglobin concentration (Zhou et al., 2013). In addition, proteomic studies showed that the aqueous extract of *Epimedium* could affect the myosin light chain (MLC)1/3, heat shock protein 27 (HSP27), glyceraldehyde-3-phosphate dehydrogenase (GAPDH), Troponin I fast (TnIf) of gastrocnemius muscle to exert their anti-fatigue effects in overtraining rats (Sun, 2014). Flavonoids are the major components of *Epimedium* with strong anti-fatigue activity: icariin and icariside I could reduce BUN and LDA levels, reduce exercise-induced oxidative stress markers (ALT, AST, and MDA) (Shang et al., 2012; Chen and Wei, 2013; Guo, 2014; Li et al., 2014). Moreover, the icariin-zinc complex showed similar effects in relieving exercise-induced fatigue (Zhang J. et al., 2021).

Cistanches Herba, also called “Desert Ginseng”, which originated from the dried fleshy stem with scaly leaves of *Cistanche deserticola* Y. C. Ma or *C. tubulosa* (Schenk) Wight (Fam. Orobanchaceae). It is another herbal medicine widely used in China and other Asian countries because of its efficacy in nourishing the kidney Yang, benefiting blood and essence, and moistening intestines to easily pass stool (Chinese Pharmacopoeia Commission, 2020). It has been reported that the ethanol extract of *C. deserticola* presented positive effects on the oxidative stress markers and the activity of antioxidant enzymes in myocardial mitochondria in exercise-induced fatigue rats, which was conducive to effectively inhibiting the oxidative damage of mitochondrial caused by excessive exercise (Gao et al., 2011; Zhou et al., 2012). The total polyphenols, glycosides, and sugar of the *Cistanche* have been demonstrated to improve the glycogen levels of the liver and muscle, increase the activity of antioxidant enzymes, and decrease the BLA, BUN, MDA, and CK levels (Wang, 2020c; Luan, 2020). The Cistanche phenylethanol glycosides and gardenia yellow pigment mixture could relieve the hypoxic exercise fatigue by improving the activities of antioxidant and metabolic enzymes, reducing the expression of apoptotic proteins, and lowering the AMP-activated protein kinase (AMPK) and NADPH oxidases 2 (Nox2) levels in hypoxic exhaustive swimming rats (Li et al., 2021a). The oligosaccharides of *C. tubulosa* have been found to exert anti-fatigue effects by reducing the accumulated harmful metabolites, maintaining the stability of hormonal levels related to the HPA axis, and enhancing the glycogen storage (Wang, 2020a). More frequently used Yin nourishing and Yang supporting TCMs showing relieving effects on exercise-induced fatigue are listed in Supplementary Table S1.

#### 4.1.2 Qi promoting and blood circulating TCMs in relieving exercise-induced fatigue

Ginseng Radix et Rhizoma (*Panax ginseng* C. A. Mey., Fam. Araliaceae), one of “the Four Pillars” of TCMs, has long been used as a medicinal and edible food for nutrient supplements and treating various diseases given its extradentary effects in reinforcing vital energy (Qi) (Chinese Pharmacopoeia Commission, 2020). Recent studies showed that the ginsenosides and ginseng polysaccharides could prolong the exercise time of different animal models: these bioactive ingredients could decrease BUN, BLA, LDA, CK, and MDA levels, increase the liver and muscle glycogen contents, enhance SOD, catalase (CAT), and GSH-Px activities, which effectively assist the body in removing oxygen free radicals produced by strenuous exercise, protecting the mitochondrial membrane from the lipid peroxidation and maintaining its integrity (Feng et al., 2010b; Jiao et al., 2021; Kong et al., 2021). The total ginsenosides could protect the excessive exercise-induced kidney injury by down-regulating the protein expression caspases-3 and increase the level of hypoxia inducible factor-1 (HIF-1α) expression (Wang et al., 2014). 20 (S)-protopanaxadiol was found to delay the exercise-induced LA accumulation by directly activating the activity of creatine kinase isoenzyme-3 (Chen F. et al., 2020). Metabonomic research indicated that ginsenoside Rh1 supplementation could reduce the α-D-glucosamine 1-phosphate content and regulate the tricarboxylic acid cycle in relieving physical fatigue (Wang, 2020b). It also has been reported that ginseng polysaccharides could restore the erythrocyte function injury caused by excessive exercise through increasing the contents of ATP, ATPase, and sialic acid in rat serum after exhaustive swimming, restoring the activities of Na^+^/K^+^-ATPase, and Ca^2+^/Mg^2+^-ATPase in a dose-dependent manner (Yang et al., 2019). In addition, ginsenosides could also reduce the contents of gamma-aminobutyric acid (GABA) and 5-HT, and increase the acetylcholine chloride (Ach), NE, and DA levels of the CNS (Feng et al., 2010a; Chen and Li, 2011), inhibit the expression of pro-inflammatory cytokines such as TNF-α in relieving inflammatory responses, and reshape the gut microbial ecosystem in alleviating exercise-induced fatigue (Zheng et al., 2021; Zhou et al., 2021).

Rhodiolae Crenulatae Radix et Rhizoma (*Rhodiola crenulata* (Hook. f. et Thoms.) H. Ohba, Fam. Crassulaceae) and its homologues from the same species are famous for their function of invigorating Qi and promoting blood circulation in both TCM and Tibetan medicine (Chinese Pharmacopoeia Commission, 2020; Wang et al., 2022b). Different extracts of *R. crenulate*, *R. rosea*, and *R. sachalinensis* have been found to reduce exercise-induced fatigue and oxidative stress by lowering the levels of BUN, MDA, CK, and LA, and increasing the activities of SOD, GSH-Px, CAT, and T-AOC to exert definite anti-exercise-induced fatigue effects (Zhou et al., 2010; Li, 2013; Jówko et al., 2018; Hou et al., 2020). In addition, the commercial *R. crenulata* oral liquid could alleviate the deficiency of energy supply by improving the levels of liver and muscle glycogen, and enhancing the activities of the Ca^2+^-ATPase and Na^+^/K^+^-ATPase to stabilize the ATP synthesis and storage. It could ameliorate exercise-induced fatigue by inhibiting mitophagy via suppressing the PINK1/Parkin signaling pathway (Hou et al., 2020). In addition, salidroside showed activity to increase the levels of the DA and NA and to decrease the 5-HT, and the 5-hydroxyindoleacetic acid (5-HIAA) contents to maintain the steady state of neurotransmitters in brain tissue of the exercise-induced fatigue mice (Gai and Zheng, 2016).

Astragali Radix (AR), the dried root of *Astragalus membranaceus* (Fisch.) Bge. var. mongholicus (Bge.) Hsiao or *A. membranaceus* (Fisch.) Bge (Fam. Leguminosae), is one of the essential and commonly used TCMs to invigorate Qi and promote Yang (Chen Z. et al., 2020; Chinese Pharmacopoeia Commission, 2020). All of the AR’s aqueous and ethanol extracts, and the total astragalosides and flavonoids exhibited an ability in improving the endogenous antioxidant capacity by reducing the levels of oxidative stress markers (BUN, MDA, LA, CK, and LDH), improving the ability of antioxidant enzymes (SOD, POD, Ca^2+^-ATPase), enhancing the regeneration of ATP and glycogen levels (Li et al., 2010; Yeh et al., 2014; Zhang G. et al., 2015). Li et al. demonstrated that the aqueous extract of AR remarkably increased the oxygen-carrying capacity and the hemoglobin (HG) content in hypoxic exhausted mice (Li et al., 2012a). Feng et al. reported that the anti-fatigue effect of total astragalosides may be linked to the improvement of hippocampal neuron injury by elevating the activities of total antioxidant capacity (T-AOC) and acetylcholinesterase (TchE), and reducing the positive cells of caspase-3 (Feng et al., 2014). Interestingly, the AR acupoint injection could alleviate exercise-induced fatigue caused by the hyperactivity of the HPA axis and maintain the balance of Th1 and Th2 cytokines (Li et al., 2012b; Li et al., 2012c). More frequently used Qi promoting and blood circulating TCMs showing relieving effects on exercise-induced fatigue are listed in Supplementary Table S2.

#### 4.1.3 Internal heat clearing TCMs in relieving exercise-induced fatigue

Portulacae Herba is the aerial part of *Portulaca oleracea L*. (Fam. Portulacaceae) which is a medicine and food homologous drug that clears internal heat and removes toxin, cools blood and stops bleeding (Chinese Pharmacopoeia Commission, 2020; Qin et al., 2020). It has been reported that the commercial extract of *P. oleracea* could extend exhaustive swimming time of mice, decrease the accumulation of BLA in skeletal muscle and the activity of LDH (Liu J. et al., 2010; Liu Z. et al., 2010). Xu and Shan illustrated the anti-fatigue effects of *P. oleracea* polysaccharides in the rotarod test and forced swimming animal models by reducing the levels of BLA and BUN, and elevating the contents of liver and muscle glycogen (Xu and Shan, 2014).

Puerariae Lobatae Radix (*Pueraria lobata* (Willd.) Ohwi, Fam. Leguminosae) has been widely planted in China and used as a medicine and food for a long time because of its healing effects in relieving muscles to expel heat, engendering liquid, and relieving thirst (Chinese Pharmacopoeia Commission, 2020; He et al., 2022). Puerarin, a kind of isoflavone glucosides, was elucidated to reduce NO content in the hippocampus, inhibit iNOS mRNA, and cGMP levels of exercise-induced fatigue rats (Guo and Xi, 2012). Besides, puerarin showed a distinctive function in improving the hemorheology of exercise-induced fatigue rats and effectively enhances the exercise capacity (Gong et al., 2012). Moreover, the puerarin supplementation remarkably decreased the apoptosis rate of hippocampal neurons by inhibiting the expression of P53 and up-regulating the Bcl-2 mRNA in swimming-exhausted rats, thus promoting fatigue recovery (Cheng and He, 2010). It also has been reported that the total flavonoids of *P. lobata* reduced skeletal muscle oxidative stress and improved the fatigue syndrome by suppressing p38 MAPK/ERK signaling pathway (Zhu, 2020). The total flavonoids of *P. lobata* showed a protective effect on brain tissue of exercise-induced fatigue rats through down-regulating the expression of β-catenin, glycogen synthase kinase 3β (GSK-3β), signaling transducer and activator of transcription 3 (STAT3), and inhibiting the accompanying inflammatory response (Jiang et al., 2020; Mo et al., 2020).

Taraxaci Herba is the entire plants of *Taraxacum mongolicum* Hand. Mazz., or *T. borealisinense* Kitam. or other sibling plants of genus *Taraxacum* (Fam. Compositae) (Chinese Pharmacopoeia Commission, 2020). It is frequently used as a heat-clearing herb because of its medicinal and edible values (Chinese Pharmacopoeia Commission, 2020). The polysaccharides and the aqueous extract of *Taraxacum* have been found to exert great anti-fatigue effects by improving the muscle and liver glycogen contents, and lowering the BLA, BUN and TG levels in exercise-induced fatigue mice (Liang et al., 2011; Zhang and Chen, 2011; Lee et al., 2012; Hu, 2014). Furthermore, the aqueous extract of *T. officinale* exhibited a potent immune-enhancing effect through increasing the synthesis and release of several cytokines (such as TNF-α, IL-12p70, and IL-10) and immunoactive mediators in the primary cultured peritoneal macrophages. The immunopotentiation of *T. officinale* may be conducive to improving the immunosuppressive state caused by exercise-induced fatigue (Lee et al., 2012).

### 4.2 Spirit and emotion regulating TCMs in relieving exercise-induced fatigue

Based on TCM theory, it is stated that exercise-induced fatigue is accompanied by the loss of spirit and emotion. Therefore, several TCMs that can calm nerves, lift spirits and emotion, such as Polygalae Radix, Acori Tatarinowii Rhizome, and Ziziphi Spinosae Semen are commonly used (Zhang et al., 2003) (Figure 1).

Polygalae Radix originated from the dried root of *Polygala tenuifolia* Willd. or *P. sibirica* L. (Fam. Polygalaceae) (Chinese Pharmacopoeia Commission, 2020). It is initially recorded in Shen Nong’s Herbal Classic, which coordinates between the heart and kidney, calms nerves, and improves intelligence (Chinese Pharmacopoeia Commission, 2020). It has been reported that the root and the aerial part of *P. tenuifolia* could distinctly prolong the swimming endurance of the load-weighted swimming mice, reduce the accumulation of metabolites, increase the glycogen contents of liver, muscle and enhance the activities of antioxidant enzymes (Wang, 2021b; Xie, 2021). In addition, 50% ethanol extract of the aerial part of *P. tenuifolia* relieves exercise-induced fatigue by ameliorating oxidative stress damage via up-regulating the protein expression of AMPK and Nrf2, the two key regulators that are involved in the energy metabolism and antioxidant response (Wang, 2021b).

Acori Tatarinowii Rhizome (*Acorus tatarinowii* Schott, Fam. Araceae) is a widely used herb in China for inducing resuscitation, which is always applied with Polygalae Radix as a medicine pair (Chinese Pharmacopoeia Commission, 2020; Song et al., 2022). Both of the aqueous extract and α-asarone of *A. tatarinowii* could reduce the levels of BUN, BLA, and MDA, and enhance the activities of SOD and TAC in skeletal muscle and hippocampus (Yan et al., 2012; Zhu et al., 2020). It also demonstrated that the *A. tatarinowii* aqueous extract and volatile oil could improve the learning and memory of the exercise-induced fatigue rats by up-regulating hippocampal ERK/CREB, and ameliorate fatigue through inhibiting exercise-induced protein expression of tryptophan hydroxylase 2 (TPH2) and synthesis of 5-HT, and increasing the protein level of 5-HT1B in the dorsal raphe. (Zhu et al., 2014; Chen et al., 2019). Moreover, cis-asarone and 5-hydroxymethyl furfural of *A. tatarinowii* showed significant activity in prolonging muscle contraction time of the isolated gastrocnemius muscle of Bufo gargarizans (Zhu et al., 2012; Zhu et al., 2013).

Ziziphi Spinosae Semen is the dried mature seeds of *Ziziphus jujuba* Mill. var. *spinosa* (Bunge) Hu ex H. F. Chou (Fam. Rhamnaceae), which has been used for centuries as a drug and nutraceutical to treat insomnia and anxiousness (Javier and Laura, 2017; Chinese Pharmacopoeia Commission, 2020). The *Z. jujuba* protein and its hydrolysates could improve the exercise capacity of loaded swimming mice given their antioxidant activity. Furthermore, the *Z. jujuba* protein and its hydrolysates could also enhance the capacities of glycogen storage and the elimination of the exercise-induced metabolites (Zhang H. Y. et al., 2021; Han et al., 2021).

## 5 Discussion

### 5.1 Major bioactive components of TCMs in relieving exercise-induced fatigue

**FIGURE 2:**
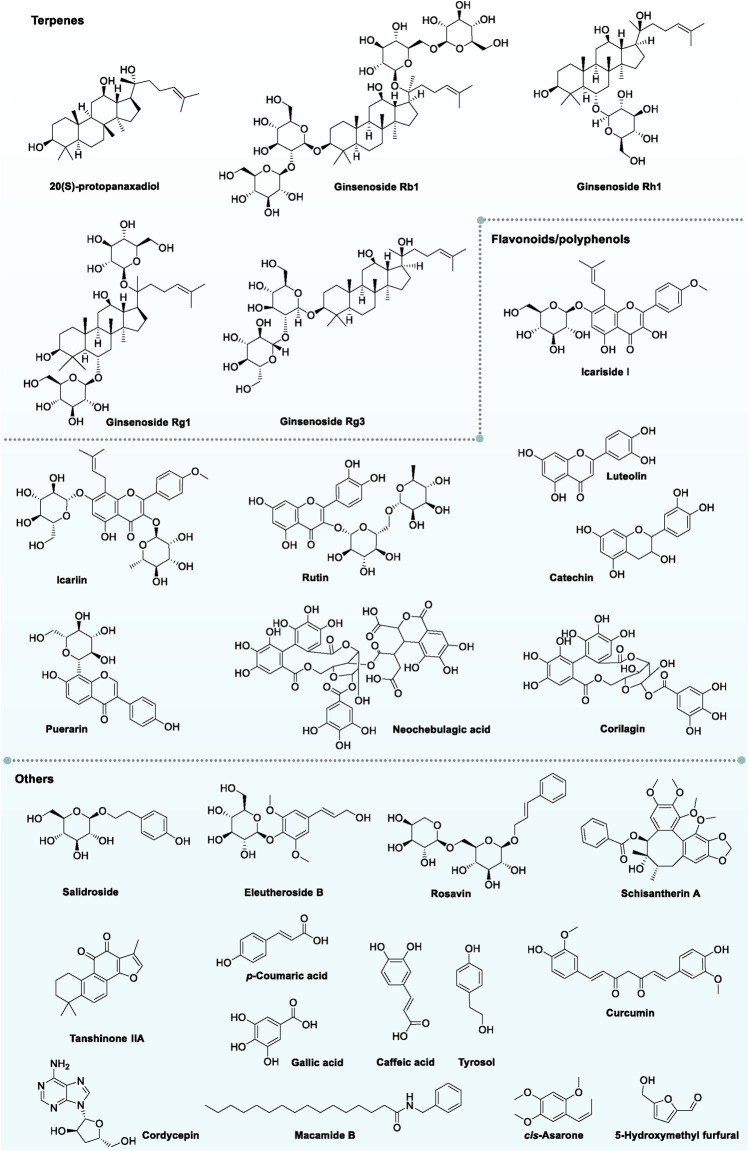
Representative chemical structures of TCMs with relieving effect on exercise-induced fatigue.

According to the aforementioned TCMs with the function of alleviating exercise-induced fatigue, polysaccharides (*Schisandra chinensis* polysaccharides, *Hippophae rhamnoides* polysaccharides, Ganoderma polysaccharides and *Polygonatum* polysaccharides, etc.), terpenes (ginsenosides, panax notoginsenosides, Astragalus saponins, etc.), flavonoids/polyphenols (puerarin, curcumin, luteolin quercetin, kaempferol, and rutin, etc.), peptides/proteins (oyster peptides, pilose antler polypeptides, ginseng oligopeptides, *P. frutescens* peptides, jujube protein and *P. lobata* protein, etc.) and other components (phenylethanoid glycosides, quinones, organic acid, and alkaloids, etc.) were illustrated to be the main bioactive components of TCMs in relieving exercise-induced fatigue. The representative chemical structures of these components are summarized in Figure 2.

### 5.2 Possible molecular mechanisms of TCMs in relieving exercise-induced fatigue

Multiple mechanisms are involved in exercise-induced fatigue, mainly the depletion of energy, metabolite accumulation, oxidative stress, inflammatory response, neurotransmitters secretion disorders, and HPA axis hypofunction (Zhou and Jiang, 2019; Ma et al., 2021). Therefore, rational and effective treatments have been taken into consideration, and the TCMs intervention has been proven to be an effective solution in help in improving exercise endurance and alleviating exercise-induced fatigue.

**FIGURE 3:**
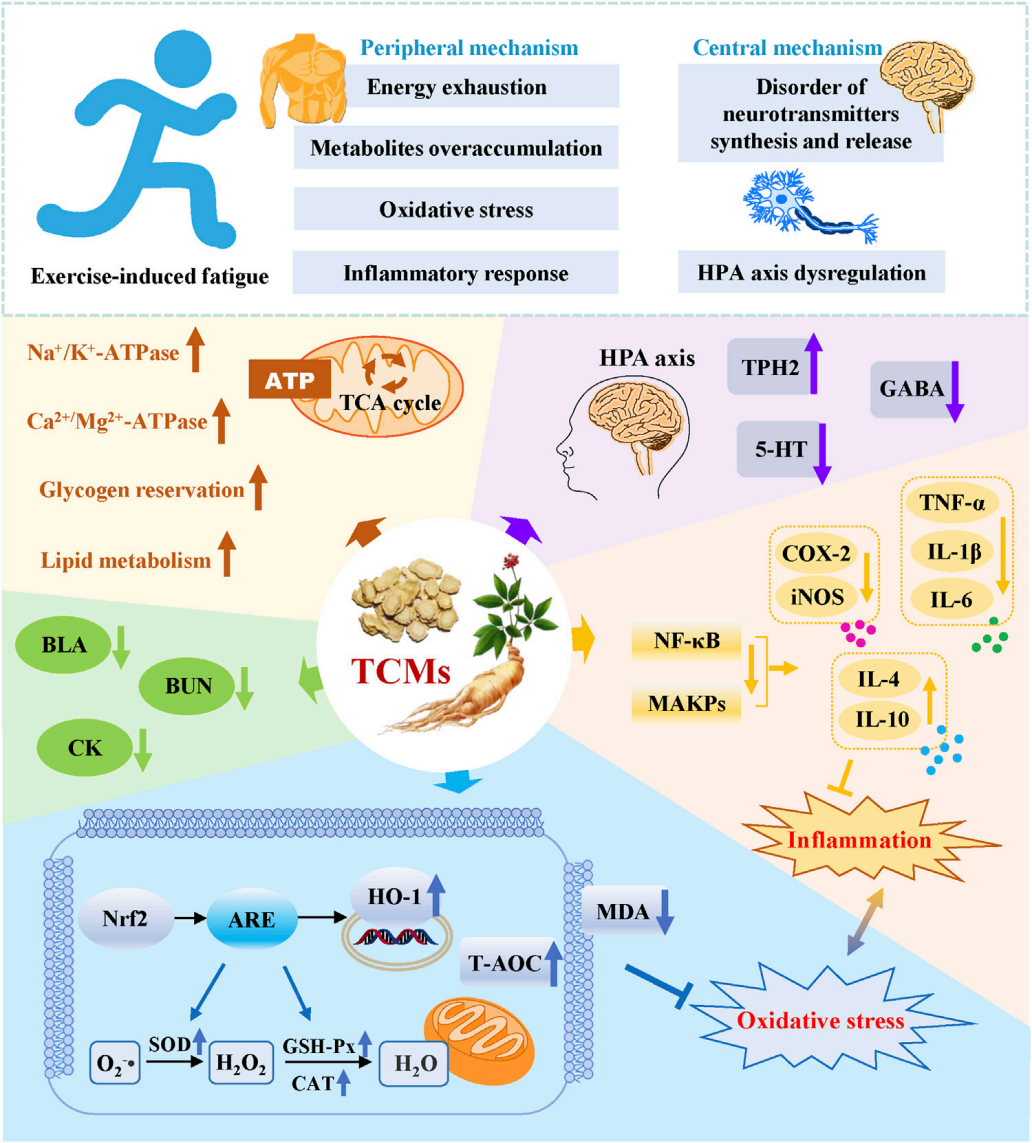
Possible molecular mechanisms of TCMs in relieving exercise-induced fatigue.

Throughout the investigations of TCMs’ ability to resist exercise-induced fatigue, active components in beating fatigue from the aspects of animal models, efficacy, and mechanisms, the possible molecular mechanisms of TCMs in relieving exercise-induced fatigue are summarized as follows: 1) The exercise-induced metabolites, such as MDA, BLA, and BUN are important indicators of physical fatigue, which can be decreased by the treatment of TCMs; 2) TCMs treatment alleviates sports fatigue-induced oxidative stress injury by enhancing the anti-oxidative enzymes via activating Nrf2-ARE antioxidative signaling pathway. Furthermore, excessive oxidative stress causes cell apoptosis, which is associated with exercise-induced fatigue. Therefore, attenuated oxidative stress by TCMs therapy also means a lower level of apoptosis and exercise-induced fatigue; 3) The glycogen of liver and muscle are the material basis in maintaining the homeostasis of blood glucose, mitochondrial oxidative phosphorylation, and glycolysis. The supplementation of TCMs increases glycogen storage, promotes lipid metabolism, and increases ATP synthesis to resist exercise-induced fatigue; 4) The excessive release of pro-inflammatory cytokines was shown to be closely related to the prolonged and high-intensity exercise. The overactivated pro-inflammatory signaling pathways such as NF-κB cascade, and these pro-inflammatory signaling-mediated productions of cytokines such as IL-6, IL-1β, and TNF-α can be effectively inhibited by the treatment of TCMs; 5) Intense exercise causes HPA axis dysregulation, which can be improved by adjusting serum corticosterone and adrenaline through TCMs conditioning (Li et al., 2012c; Wang, 2020a); 6) The disorder of brain neurotransmitters (5-HT, DA, and NE) synthesis and release often occurs because of the prolonged exercise. TCMs therapy has been shown to inhibit the high concentrations of 5-HT and GABA, elevate the expression of TPH2, and enhance the protein expression of 5-HT1B receptor in fatigued animal models, suggesting the regulatory role of TCMs in neurotransmitter disorders (Feng et al., 2010a; Zhu et al., 2014; Gai and Zheng, 2016; Chen et al., 2019) (Figure 3).

### 5.3 Limitations of the current research on TCMs in relieving exercise-induced fatigue

Presently, although TCMs hold considerable promise in relieving exercise-induced fatigue, the limitations of these studies are still significant. Most of the study outcomes are based on animal experiments with various models and evaluation indexes, which are unfavorable to the comprehensive comparison and evaluation of different TCMs. Besides, human clinical trials are still insufficient and the majority of them are emphasized on the detection of simple biomarkers, the subjective feelings and evaluations of the subjects (Bach et al., 2016). Currently, the most studied anti-fatigue TCMs are also the frequently used tonic medicines in clinical applications, such as *P. ginseng*, *Cordyceps sinensis*, and *Ganoderma lucidum* (Zhao, 2019; Li, 2020) (Supplementary Table S3). However, there is insufficient clinical evidence to support the significant effect of these TCMs in relieving exercise-induced fatigue and improving physical performance.

The following problems, such as limited number of subjects, uneven distribution of genders, and various assessment method generally exist in these clinical studies, which result in the lack of strict objectivity, repeatability and difficulty in the comparison of the anti-fatigue effects of these TCMs. Therefore, the human clinical trials would pay more attention in the future and some suggestions are proposed here: 1) Randomized, double-blind, placebo-controlled studies are needed in clinical trials; 2) Adequate subject numbers and balanced sex ratio are important for the objective results; 3) Standard assessment and evaluation system should be gradually established to acquire the fully proved results; 4) Reasonable measurement methods and proper TCMs selection are supposed to be taken based on the personal conditions and symptoms, according to the viewpoint of syndrome differentiation and treatment in TCM theory; 5) Mechanisms deserve further research at cellular and molecular levels.

### 5.4 Cautions in the herb-induced liver injury

With the increased applications and consumptions of TCMs, the risk of herb-induced liver injury (HILI) has been concerned (Kaplowitz, 2018). TCMs such as Polygoni Multiflori Radix, Cassiae Semen, Aloe and Toosendan Fructus are typical hepatotoxic medicines (Chau et al., 2011; Danan and Teschke, 2016; He et al., 2019; Ballotin et al., 2021). It has been demonstrated that some alkaloids, terpenes, anthraquinones exhibit potential hepatotoxicity. In the present review, most of the exercise-induced fatigue relief TCMs are mild tonic medicines and no significant hepatotoxicity was observed, only except Polygoni Multiflori Radix and Cassiae Semen. The processed Polygoni Multiflori Radix (stewing or steaming with the decoction of black soybean) are commonly used in clinical practice, which effectively reduced its hepatotoxicity (Tu et al., 2015). Besides, the injury effect on liver is only observed in a long term and high dose application of Cassiae Semen (Huang et al., 2022). Generally, these anti-fatigue TCMs are used in prescriptions, thus the reasonable dosage and the compatibility between TCMs can neutralize these side effects. However, unlike synthetic drugs, TCMs are integrations of multiple ingredients, the dose and course of treatment, drug interactions may all be risks of HILI. Therefore, potential HILI risk assessment focused on hepatotoxicity in the early stage of drug discovery and clinical application is indispensable. All in all, strictly control the dosage and duration of the potential hepatotoxic TCMs according to gender, individual constitution and age of the patients, is the effective way to prevent HILI.

## 6 Conclusion

Multi-ingredients, multi-targets and multi-pathways regulation are the most important characteristics of TCMs, which alleviated exercise-induced fatigue from multi-aspects. According to the holistic concept and syndrome differentiation of the TCM theory, viscera tonic TCMs are the most widely used procedure in clinical applications. Most of these TCMs are edible medicines and are usually applied in prescriptions. It is generally believed that exercise-induced fatigue belonged to physiological fatigue category, which can recover automatically. The main purpose of TCMs supplementation is to help speed up the recovery of exercise-induced fatigue, increase the fatigue tolerance of the human body, or improve sports performance.

Although current research results are mostly acquired from animal models, the clinic trials are still insufficient, and a very few TCMs have been reported to possess potential hepatotoxicity. These findings still offer great reference value, and the significant efficacy in relieving exercise-induced fatigue is impossible to ignore. In conclusion, the exercise-induced fatigue relieving effects of TCMs are clear, which tend to be the potential therapeutic strategies for athletes, military personnel, and manual laborers. We hope the present review will provide scientific evidence for a better understanding of the relieving effects of TCMs on exercise-induced fatigue, and offer guidance to promote the further research and development of specific products for the specific fatigue group.

## Data Availability

The original contributions presented in the study are included in the article/Supplementary Material; further inquiries can be directed to the corresponding authors.
