# Supplementary material for: The use of traditional Chinese medicines in relieving exercise-induced fatigue
Source: Front Pharmacol. 2022 Jul 22;13:969827. doi: 10.3389/fphar.2022.969827 (PMC9353218; doi:10.3389/fphar.2022.969827)
Supplement: Supplementary file 1 [file DataSheet1.docx]

Supplementary Material

# Supplementary Tables

**Table S1** Representative Yin nourishing and Yang supporting TCMs in reliving exercise-induced fatigue.

| **No.** | **TCM** | **Source** | **Mechanism** | **Animal model** | **Bioactive component/ part** | **Reference** |
| --- | --- | --- | --- | --- | --- | --- |
| 1 | Cordyceps | *Cordyceps sinensis* (BerK.) Sacc.  (now known officially *Ophiocordyceps sinensis*)  (Clavicipitaceae) | Liver and muscle glycogen↑, AMPK↑, PGC-1α↑, PPAR-δ↑, IgG↑, IgA↑, serum C3, C4 and CH50↑, BLA↓, MDA↓, activating NRF-2-ARE pathway. | Weight-loaded swimming test/ without weight-loaded swimming test | Oral liquid of *C. sinensis*/ air-dried power of mycelium/ aqueous extracts of fruit body/ aqueous extracts/ polysaccharide | Li (2020a); Kumar et al. (2011); Luan et al. (2013); Yan et al. (2012); Huang (2019) |
| 2 | Cordyceps Militaris | *Cordyceps militaris* (L.ex Fr.) Link.  (Clavicipitaceae) | ATP↑, liver and muscle glycogen↑, SOD↑, CAT↑, GSH-Px↑, hippocampal Glu↑, ACh↑, ALT↓, AST↓, LDH↓, BLA↓, CK↓, GABA↓, 5-HT↓, activating AMPK and AKT/mTOR pathways and regulating serum hormone level. | Grip strength test/ weight-loaded swimming test/ forced running and rotating rod and weight-loaded forced swimming tests/ treadmill test | Ethyl acetate extract/ extruded product of cereal grains mixed with *C. militaris*/ fruit body aqueous extract/ cordycepin/ | Choi et al. (2020); Zhong et al. (2017); Song et al. (2015); Zhang (2021) |
| 3 | Cervi Cornu Pantotrichum | *Cervus elaphus* Linnaeus  *C. nippon* Temminck  (Cervidae) | Liver and muscle glycogen↑, SOD↑, GSH-Px↑, BLA↓, BUN↓, MDA↓ and promoting free radical scavenging activity. | Weight-loaded swimming test/ rotating rod test/ | *C. elaphus* composition/ freeze-dried powder of sika deer antler blood/ freeze-dried sika deer antler/ polypeptide/ aqueous extract/ liposoluble components | Liang et al. (2020); Qin et al. (2019); Jin (2015); Hu et al. (2015); Zhang et al. (2011a); Zhang (2010) |
| 4 | Hippocampus | *Hippocampus kelloggi* Jordan et Snyder (Syngnathidae) | LA↓ | Rotating rod test | Power of *H. kelloggi* | Tang (2019) |
| 5 | Ostreae Concha | *Ostrea rivularis* Gould  (Ostreidae) | GSH-Px↑, Mn-SOD↑, PGC-1α↑, TFAM↑, liver glycogen↑, openness of MPTP↓, BLA↓, BUN↓, LDH↓, CK↓ | Weight-loaded swimming test | Peptide/ meat hydrolysate | Luo et al. (2021); Wang (2020); Tao et al. (2020); Ji et al. (2010) |
| 6 | Rehmanniae Radix Praeparata | *Rehmannia glutinosa* Libosch.  (Scrophulariaceae) | Liver glycogen↑, BLA↓, BUN↓ | Weight-loaded swimming test | Polysaccharide | Tan et al. (2012) |
| 7 | Polygonati Rhizoma | *Polygonatum sibiricum* Red.  *P. cyrtonema* Hua  *P. kingianum* Coll. et Hemsl.  (Asparagaceae) | Ca^2+^/Mg^2+^-ATP↑, liver and muscle glycogen↑, GSH-Px↑, DA↑, SOD↑, T-AOC↑, MDH↑, BUN↓, BLA↓, MDA↓, lipid peroxidation↓, 5-HT↓, SDH↓ | Weight-loaded or exhaustive swimming test | Polysaccharide/ aqueous extract/ flavonoids/saponin | Zhang et al. (2021); Chen et al. (2021); Guo et al. (2021); Li (2020b); Yang et al. (2019); Fu (2019); Li (2014b); Wang et al. (2014); Shi et al. (2011); Du et al. (2021); Chen (2012) |
| 8 | Hippophae Fructus | *Hippophae rhamnoides* L.  (Elaeagnus) | Liver and muscle glycogen↑, BLA↓, BUN↓, heart rate↓, 5-HT↓, NEFA↓, NH_3_-N↓ | Exhaustive or weight-loaded swimming test/ 20 km race test (Mongolian horse) | Polysaccharides/ aqueous extract/ flavonoids/ seed meal protein | Liu et al. (2012); Su (2020); Wang et al. (2020); Dang (2021); Zhang et al. (2011b); Jiao (2010) |
| 9 | Lycii Ruthenici Fructus | *Lycium ruthenicum* Murr.  (Solanaceae) | Blood sugar↑, testosterone↑, SOD↑, CAT↑, GSH-Px↑, liver and muscle glycogen↑, BLA↓, MDA↓, LPO↓, ROS↓, BUN↓, enhancing free radical scavenging capacity, and protecting exercise-induced myocardial injury. | Exhaustive or weight-loaded swimming test/ gastrocnemius of toad test | Aqueous extract | Xing and Ke (2019); Ma (2019); Yang et al. (2018a); Cao et al. (2019); Yang et al. (2018b); Cao et al. (2017) |
| 10 | Eucommiae Cortex | *Eucommia ulmoides* Oliver  (Eucommiaceae) | Liver and muscle glycogen↑, blood sugar↑, HGB↑, insulin↑, growth hormone↑, BU↓, LA↓ | Endurance treadmill test | Commercial extract | Wang et al. (2013) |
| 11 | Pseudostellariae Radix | *Pseudostellaria heterophylla* (Miq.) Pax.  (Caryophyllaceae) | HGB↑, CAT↑, SOD↑, GSH-Px↑, MDA↓, BLA↓ | Exhaustive swimming test | Polysaccharide | Chen et al. (2013) |
| 12 | Polygonati Odorati Rhizoma | *Polygonatum odoratum* (Mill.) Druce  (Asparagaceae) | GSH-Px↑, muscle glycogen↑, SOD↑, CAT↑, GSH-Px↑, LDH↓, LA↓, CK↓, BUN↓, MDA↓ | Weight-loaded swimming test | Polysaccharide | Niu (2018) |
| 13 | Cuscutae semen | *Cuscuta chinensis* Lam.; *C. australis* R.Br.  (Convolvulaceae) | Liver and muscle glycogen↑, SOD↑, GSH-Px↑, MDA↓, BLA↓ | Weight-loaded swimming test | Aqueous extract | Guo et al. (2013); Yin et al. (2012) |
| 14 | Schisandrae Chinensis Fructus | *Schisandra chinensis* (Turcz.) Baill.  (Magnoliaceae) | ATPase↑, Liver and muscle glycogen↑, SOD↑, GSH-Px↑, Nrf2↑, HO-1↑, creatine↓, LDH↓, CK↓, PARP↓, Caspase-3↓, NT↓, 4HNE↓, iNOS↓, BLA↓, BUN↓, ROS↓, MDA↓, 8-OHdG↓, liver Keap1↓, improving the phagocytic index and phagocytic coefficient K value of a value. | Forced swimming or weight-loaded test/ sit-in test/ rotating rod test/ forelimb grip test | ethanol extract/ organic acid/ polysaccharide/ lignans/ schisantherin A | Kim et al. (2018); Li et al. (2014); Fang et al. (2022); Wang (2012); Zhang and Xu (2011); Huang et al. (2019); Zhang et al. (2020b) |
| 15 | Acanthopanacis Senticosi Radix Et Rhizoma Seu Caulis | *Acanthopanax senticosus* (Rupr.et Maxim.) Harms  also known as *Eleutherococcus senticosus* (Araliaceae) | Liver and muscle glycogen↑, skeletal muscle fatty acid and β-oxidation↑, SOD↑, brain eNOS↑, Na^+^/K^+^-ATPase↑, Ca^2+^/Mg^2+^-ATPase↑, Glu/GABA↑, Nrf2↑, HO-1↑, BUN↓, BLA↓, LDH↓, TG↓, MDA↓, ROS↓, Keap1↓, ALT↓, AST↓, regulating the expression of Keap1/Nrf2/ARE signaling pathway-related factors. | Forced or/and weight-loaded swimming test/ treadmill running and swimming tests/ rotating rod test/ treadmill running test | Seed ethanol extract/commercial extract/ liposoluble fraction/ stem bark aqueous extract/ n-butanol fraction and eleutheroside E/ eleutheroside B | Li (2018); Sumiyoshi and Kimura (2016); Huang et al. (2011a); Zhang et al. (2010); Kato et al. (2013); Huang et al. (2011b); Zhang et al. (2020a); Wu et al. (2013) |
| 16 | Cnidii Fructus | *Cnidium monnieri* (L.) Cuss. (Umbelliferae) | Liver and muscle glycogen↑, HGB↑, serum testosterone↑, BUN↓ | Weight-loaded swimming test | Aqueous extract | Deng et al. (2013) |
| 17 | Astragali Complanati Semen | *Astragalus complanatus* R. Br.  (Leguminosae) | Hippocampal and diencephalon aliphatic EAA↑, Glu/GABA↑, Glu↑, ASP↑, 5-HIAA↑, GABA-T and SSADH↑, liver and muscle glycogen↑, Hippocampal and diencephalon aliphatic IAA↓, 5-HT↓, 5-HTP↓, GAD_67_↓, BUN↓, BLA↓, f-Trp/BCAA↓, AAA/BCAA↓, improving cerebral hypoxia, excite hippocampal region, and maintaining the normal structure and function of cells. | Endurance treadmill test | Commercial granules of *A. complanatus* | Xiong et al. (2017); Liu et al. (2017a; 2017b); Yang (2013); Zhu (2010); Yang and Xiong (2010) |
| 18 | Ligustri Lucidi Fructus | *Ligustrum lucidum* Ait.  (Oleaceae) | GSH-Px↑, MDA↓, CK↓, LDH↓ | Treadmill test | Commercial extract | Qi and Xiong (2011; 2013) |
| 19 | Gynostemmatis Pentaphylli Herba | *Gynostemma pentaphyllum* (Thunb.) Mak.  (Cucurbitaceae) | HGB↑, liver and muscle glycogen↑, SOD↑, liver Bcl-2↑, GSH-Px↑, CAT↑, BLA↓, BUN↓, MDA↓, liver Bax↓, and enhancing free radical scavenging activity. | Weight-loaded or exhaustive swimming test | Polysaccharides/ ethanol extract/ gypenosides | Shan and Shi (2014); Long (2010); Su and Qi (2016); Ding et al. (2010)(Ding, 2010 #2765;龙碧波, 2010 #2812;宿彦峰, 2016 #2800) |
| 20 | Eucommiae Folium | *Eucommia ulmoides* Oliver  (Eucommiaceae) | Liver and muscle glycogen↑, BLA↓, SUN↓ | Weight-loaded swimming test/ gastrocnemius of toad test | Polyphenol/ polysaccharide | Liu et al. (2013); Xia and Pu (2010) |
| 21 | Morindae Officinalis Radix | *Morinda officinalis* How  (Rubiaceae) | GSH-Px↑, liver glycogen↑, SOD↑, MDA↓, LA↓, BUN↓, improving the activity of mitochondrial antioxidant enzyme. | Weight-loaded swimming test | Polysaccharide/ aqueous extract | Liang et al. (2018); Long et al. (2013); Zhu et al. (2013) |
| 22 | Curculiginis Rhizoma | *Curculigo orchioides* Gaertn.  (Amaryllidaceae) | SOD↑, GSH-Px↑, IGF-1↑, mTOR mRNA↑, MDA↓ | Exhaustive weight-loaded swimming test/ single contraction tension test | Aqueous extract | Jiang (2014); Li (2014a) |
| 23 | Saussureae Involucratae Herba | *Saussurea involucrata* (Kar. et Kir.) Sch.-Bip.  (Compositae) | PGC-1α↑, SIRT1 mRNA and protein↑, BUN↓, BLA↓, MDA↓, activating the MAPK/ERK1/2 signaling pathway. | Weight-loaded or forced swimming test | Rutin/ cell cultures of *Saussurea involucrate* | Su et al. (2014); Liu (2012) |
| 24* | Polygoni Multiflori Radix | *Polygonum multiflorum* thunb.  (Polygonaceae) | Liver glycogen↑, SOD↑, BLA↓, MDA↓ | Weight-loaded swimming test | Polysaccharide | Cheng et al. (2016) |
| 25* | Cassiae Semen | *Cassia tora* L.  *C. obtusifolia* L.  (Leguminosae) | GSH-Px↑, SOD↑, RMR↓, MDA↓, BUN↓, CK↓, TG↓, BLA↓, LDH↓ | Without weight-loaded/ weight-loaded swimming test | Aqueous extract/ polysaccharides | Ma et al. (2019); Kang et al. (2021) |

*****Hepatotoxicity has been reported.

**Table S2** Representative Qi promoting and blood circulating TCMs in reliving exercise-induced fatigue.

| **No.** | **TCM** | **Source** | **Mechanism** | **Animal model** | **Bioactive component/part** | **Reference** |
| --- | --- | --- | --- | --- | --- | --- |
| 1 | Panacis Quinquefolii Radix | *Panax quinquefolium* L.  (Araliaceae) | GSH-Px↑, SDH↑, Na^+^/K^+^-ATPase↑, Ca^2+^/Mg^2+^-ATPase↑, liver and muscle glycogen↑, NRF-1 and TFAM mRNA↑, skeletal muscle ATP↑, T-SOD↑, blood sugua↑, BLA↓, MDA↓, BUN↓ | Forced swimming test/ Rotating rod and pole climbing and treadmill test/ swimming test/ exhaustive swimming test under simulated plateau environment | Protein/ small-molecule oligopeptides/ultrafine powder/ ginsenoside Re, Rb1 and Rb2/ ethanol extract | Qi et al. (2014); Li et al. (2018); Bai et al. (2020); Lin et al. (2019); Zhou et al. (2013) |
| 2 | Ganoderma | *Ganoderma lucidum* (Leyss.ex Fr.) Karst.  (Ganodermataceae) | Liver and muscle glycogen↑, GSH-Px↑, SOD↑, CAT↑, BLA↓, BUN↓, SUN↓, CK↓, MDA↓, AKP↓ | Exhaustive or weight-loaded swimming test | Polysaccharide/ ganoderma lucidum spore powder | Hu et al. (2012); Cai et al. (2021); Zhao et al. (2014); Wang et al. (2019) |
| 3 | Angelicae Sinensis Radix | *Angelica sinensis* (Oliv.) Diels  (Umbelliferae) | Liver and muscle glycogen↑, BLA↓, BA↓, CK↓ | Forelimb grip test and swimming exercise training and exhaustive swimming test | Commercial powder product | Yeh et al. (2014) |
| 4 | Salviae Miltiorrhizae Radix Et Rhizoma | *Salvia miltiorrhiza* Bge.  (Labiatae) | Liver and muscle glycogen↑, TG↓, CK↓, LDH↓, BUN↓, MDA↓, IL-6↓, TNF-α↓, hsCRP↓, regulating energy metabolism, oxidant-antioxidant balance and the endogenous metabolites in the exercising muscle, protecting myocardium. | Weight-loaded or exhaustive swimming test | Commercial total extract/ ethanol extract/ tanshinone ⅡA | Wang et al. (2021); Gao et al. (2011); Zhang et al. (2012); Liu and Ling (2012) |
| 5 | Codonopsis Radix | *Codonopsis tangshen* Oliv.  (Campanulaceae) | Liver glycogen↑, PGC-1α↑, BLA↓, BUN↓ | Weight-loaded swimming test | Ethanol extract | Shang and Li (2018) |
| 6 | Phyllanthi Fructus | *Phyllanthus emblica* L.  (Euphorbiaceae) | Liver glycogen↑, SOD↑, GSH-Px↑, NO↑, BLA↓, MDA↓ | Exhaustive swimming test under simulated plateau environment/ exhaustive or weight-loaded swimming test | Ethanol extract/ aqueous and ethanol merged extracts | Zhang et al. (2011c); Nie et al. (2014) |
| 7 | Paeoniae Radix Alba | *Paeonia lactiflora* Pall.  (Ranunculaceae) | BLA↓, BUN↓ | Exhaustive and weight-loaded swimming test | Polysaccharide | Li (2021) |
| 8 | Perillae Fructus | *Perilla frutescens* (L.) Britt.  (Labiatae) | Liver and muscle glycogen↑, BLA↓, BUN↓ | Weight-loaded swimming test | Peptides | Wei et al. (2021) |
| 9 | Phedimus Aizoon Herba | *Sedum aizoon* L.  (Crassulaceae) | Liver and muscle glycogen↑, blood sugar↑, SOD↑, GSH-Px↑, BUN↓, BLA↓, MDA↓, blood perfusion index↓, cortisol↓, CK↓, LAC↓, LDH↓ | Weight-loaded swimming test/pole climbing test | Pressed juice | Ding (2019); Ren (2020) |

**Table S3** Clinical trials of TCMs in reliving exercise-induced fatigue.

| **No.** | **TCM** | **Subject** | **Study design and duration** | **Outcome** | **Measurement tool** | **Reference** |
| --- | --- | --- | --- | --- | --- | --- |
| 1 | *P. ginseng*  (Enzyme-modified ginseng extract) | 52 healthy subjects | Randomized, double-Blind, placebo-controlled crossover trial.  Enzyme-modified ginseng extract or placebo were administered to each group for 4 weeks. | Significant reduction in the VAFS scores.  No fatal adverse effects. | VAFS and RPFS scores | Lee et al. (2016) |
| 2 | *P. ginseng*  (20-Ο-β-D-glucopyranosyl-20(S)-protopanaxadiol; or Compound K) | 10 healthy females and nine healthy melas | Double-blind, placebo-controlled trial.  High dose ginseng (960 mg/day), low dose ginseng (160 mg/day) and placebo (0 mg/day).  Three-cycle testing scheme: Each 14-day supplementation cycle was separated by a 7-day washout period. | Significantly improved perceived exercise-exertion, muscular pain/ soreness and neuromuscular fatigue following an acute bout of intense resistance exercise | Acute bout of resistance exercise, quick board reaction time tests, ballistic jump power, questionnaires, perceptual measures | Caldwell et al. (2018) |
| 3 | *C. sinensis*  (Extract of *C. sinensi*: polysaccharide) | 20 long-distance runners (10 males and 10 females) | Randomized-controlled trial.  Every morning and evening, the male and female long-distance runners in the experimental group each took a cup of warm water containing 1.5 g Cordyceps polysaccharide, 4 consecutive weeks. | HGB↑, BLA↓, BUN↓, CK↓, heart rate↓ | HGB, BLA, BUN, CK, heart rate | Zhao (2019) |
| 4 | *G. lucidum*  (*G. lucidum* polysaccharide oral liquid) | 60 cyclists (39 males and 21 females) | Randomized, placebo-controlled trial.  10 mL *G. lucidum* polysaccharide oral liquid for the test group twice a day for 6 days, suspension of medication for one day, last for 90 days. | Muscle content↑, total work↑, exercise heart rate↑, HGB↑, volume ratio of red blood cells↑, IgA↑, IgG↑, IgM↑, body fat rate↓, heart rate↓, BLA↓ | Heart rate, body weight, muscle level, body fat rate, IgA、IgM、IgG | Li (2020c) |
| 5 | *S. aizoon*  (Juice of *S. aizoon*) | 39 healthy college students (males) trained with cycling until fatigue | Single blind, randomized-controlled trial.  Water for blank group, Red Bull positive control group and *S. aizoon* juice for experimental group (5 mL/day), once every three days and last for 14 days. | Prolong exercise duration, improve exercise loads,  HGB↑, cortisol↓, PI2↓, CK↓ | Physiological and biochemical indexes evaluation | Ren (2020) |

**References**

Bai, X., Liu, H., Song, Y., Lv, G., Ye, H., Lin, H., et al. (2020). Anti-fatigue effect and acute toxicity of American ginseng ultrafine powder based on microbial diversity. *Mod. Food Sci. Technol.* 36, 1-8. doi: 10.13982/j.mfst.1673-9078.2020.4.001.

Cai, M., Xing, H., Tian, B., Xu, J., Li, Z., Zhu, H., et al. (2021). Characteristics and antifatigue activity of graded polysaccharides from Ganoderma lucidum separated by cascade membrane technology. *Carbohydr. Polym.* 269, 118329. doi: 10.1016/j.carbpol.2021.118329.

Caldwell, L.K., Dupont, W.H., Beeler, M.K., Post, E.M., Emily C. Barnhart, E. C., Hardesty, V. H. et al (2018). The Effects of a Korean Ginseng, GINST15, on perceptual effort, psychomotor performance, and physical performance in men and women. *J. Sports Sci. Med*. 17, 92-100.

Cao, R., Yang, X., Luo, H., Qiu, D., and Wang, X. (2019). Effect of Tibetan *Lycium ruthenicum* Murr. on the contractile function of isolated toad gastrocnemius muscle. *Chin. J. Clin. Pharmacol.* 35, 261-264. doi: 10.13699/j.cnki.1001-6821.2019.03.019.

Cao, R., Yang, X., Sun, M., Zhang, Y., and Luo, H. (2017). Relieving effects of *Lycium ruthenicum* Murr. on exercise fatigue in mice. *Chin. J. Clin. Pharmacol.* 33, 351-354. doi: 10.13699/j.cnki.1001-6821.2017.04.017.

Chen, X.N. (2012). Effect of *Polygonatum* extract on energy metabolism of quadriceps in swimming mice*.* [master’s thesis]. [Qufu (SD)]: Qufu Normal University.

Chen, Y., Hu, H., Feng, G., Wei, T., Li, X., Yu, L., et al. (2021). Anti-fatigue and anti-oxidant effects of crude and processed *Polygonatum cyrtonema* on exhaustive swimming mice. *Pharmacol. Clin. Chin. Mater. Med.* 37**,** 92-96. doi: 10.13412/j.cnki.zyyl.2021.02.011.

Chen, Z., Li, S., Wang, X., and Zhang, C.L. (2013). Protective effects of Radix Pseudostellariae polysaccharides against exercise-induced oxidative stress in male rats. *Exp. Ther. Med.* 5, 1089-1092. doi: 10.3892/etm.2013.942.

Cheng, K., Zhang, H.J., Chen, Y.L., Zhan, J.P., Zhang, Y.T., Xie, Z.L., et al. (2016). Anti-fatigue effect and mechanism of *Polygonum multiflorum* polysaccharide in mice. *Chin. J. Gerontol.* 36, 6054-6055. doi: 10.3969/j.issn.1005-9202.2016.24.003.

Choi, E., Oh, J., and Sung, G.H. (2020). Beneficial effect of *Cordyceps militaris* on exercise performance via promoting cellular energy production. *Mycobiology* 48, 512-517. doi: 10.1080/12298093.2020.1831135.

Dang, L. (2021). Optimization of protein extraction process and anti-fatigue activity of sea buckthorn seed meal. *Chin. Condiment* 46, 133-136. doi: 10.3969/j.issn.1000-9973.2021.11.025.

Deng, Y., Cao, J., Zhou, H., and Zhen, M. (2013). Effect of *Fructus cnidii* on testosterone content, substance metabolism and exercise capacity in rats receiving exercise training. *Chin. J. Exp. Tradit. Med. Formulae* 19, 262-266. doi: 10.11653 /syfj2013190262.

Ding, Q. (2019). Experimental study on anti-exercise-induced fatigue of Jingtian Panax notoginseng. [master’s thesis]. [Beijing (BJ)]: Capital University of Physical Education and Sports.

Ding, Y.J., Tang, K.J., Li, F.L., and Hu, Q.L. (2010). Effects of gypenosides from *Gynostemma pentaphyllum* supplementation on exercise-induced fatigue in mice. *Afr. J. Agric. Res.* 5, 707-711. doi: 10.5897/AJAR10.002.

Du, X., Liang, Z., Xia, Y., Zhang, X.Q., Zhang, W.C., Han, J.L., et al. (2021). Comparative study on anti-exercise fatigue effects of different extracts from raw materials of *Polygonatum cyrtonema* Hua and its processed products. *J. Anhui Agric. Univ.* 48, 26-30. doi: 10.13610/j.cnki.1672-352x.20210319.015.

Fang, J., Liu, J., Jiang, Y., Jing, S., Wang, C., Sun, J., et al. (2022). Effect of polysaccharides from root, stem, leaf and fruit of *Schisandra chinensis* on exercise endurance in d-galactose-induced aging mice. *Chin. J. Exp. Trad. Med. Formula* 28, 102-109. doi: 10.13422/j.cnki.syfjx.20220825.

Fu, H.L. (2019). Preliminary study on preparation process and anti-fatigue effect of *Polygonatum kingianum* crude polysaccharide tablets*.* [master’s thesis]. [Kunming (YN)]: Yunnan University of Chinese Medicine.

Gao, L., Yang, L., and Huang, Z. (2011). Effect of total extracts from *Salvia miltiorrhiza* on sports fatigue. *Chin. J. Basic. Med. Tradit. Chin. Med.* 17**,** 966-967+970.

Guo, A., Cao, J., Zhu, J., and Zhou, H. (2013). Effects of *Cuscuta chinensis* on ability of resistance and free radical in brain tissue in exercise-induced fatigue rats. *Chin. J. Exp. Trad. Med. Formula* 19, 274-277. doi: 10. 11653/syfj2013090274.

Guo, N., Wang, H.Y., Chen, R., Gao, L.X., Liao, Z.S., Luo, X.Q., et al. (2021). Optimization of solid-state fermentation conditions of *Polygonatum* by *Bacillus subtilis* and its quality and pharmacological activity. *Chin. Tradit. Pat. Med.* 43, 464-468. doi: 10.3969/j.issn.1001-1528.2021.02.032.

Hu, J., Yan, F., Zhang, Z., and Lin, J. (2012). Evaluation of antioxidant and anti-fatigue activities of *Ganoderma lucidum* polysaccharides. *J. Anim. Vet. Adv.* 11, 4040-4044. doi: 10.3923/javaa.2012.4040.4044.

Hu, T., Liu, Y., Tao, R., Su, F., Zhang, J., Li, Q., et al. (2015). Study on antifatigue mechanism of pilose antler polypeptides. *J. Jilin Agric. Univ.* 37**,** 469-476. doi: 10.13327/j.jjlau.2015.2399.

Huang, L., Geng, Y., Yan, J., Wang, Q., and Wang, Y. (2019). The Effects of *Schisandra* lignans on the negative emotion and behavior of rats with sleep deprivation. *Food Res. Dev.* 40, 55-58. doi: 10.3969/j.issn.1005-6521.2019.07.009.

Huang, L.Z., Huang, B.K., Liang, J., Zheng, C.J., Han, T., Zhang, Q.Y., et al. (2011a). Antifatigue activity of the liposoluble fraction from *Acanthopanax senticosus*. *Phytother. Res.* 25, 940-943. doi: 10.1002/ptr.3346.

Huang, L.Z., Huang, B.K., Ye, Q., and Qin, L.P. (2011b). Bioactivity-guided fractionation for anti-fatigue property of *Acanthopanax senticosus*. *J. Ethnopharmacol.* 133, 213-219. doi: 10.1016/j.jep.2010.09.032.

Huang, X. (2019). Effects of *Ophiocordyceps sinensis* polysaccharide extract on relieving acute exercise. fatigue in mice. *Edible Fungi China* 38, 43-45+49. doi: 10.13629/j.cnki.53-1054.2019.12.012.

Ji, H., Miao, J., Shao, H., and Zhang, C. (2010). Study on the nutritional components and the anti-fatigue effects of hydrolysates of *Ostrea rivularis* meat. *Food Sci. Technol.* 35, 70-73. doi: 10.13684/j.cnki.spkj.2010.02.057.

Jiang, S.S. (2014). Experimental study on the relationship between dose-effect and time-effect of *Curculigo orchioides* on exercise ability in rats. [master’s thesis]. [Qufu (SD)]: Qufu Normal University.

Jiao, Y. (2010). Studies on separation, purification and bioactivity of flavonoids from seabuckthorn*.* [dissertation]. [Harbin (HLJ)]: Northeast Forestry University.

Jin, M.Y. (2015). Study on differential proteomics and anti-fatigue activity of pilose antler with different processing technologies. [master’s thesis]. [Beijing (BJ)]: Beijing University of Chinese Medicine.

Kang, C., Liu, Y., Chi, A., and Zhang, Z. (2021). The anti-fatigue potential of water-soluble polysaccharides of Semen Cassiae on BALB/c mice. *Cell. Mol. Biol.* 67, 148-154. doi: 10.14715/cmb/2021.67.2.23.

Kato, M., Kurakane, S., Nishina, A., Park, J., and Chang, H. (2013). The blood lactate increase in high intensity exercise is depressed by *Acanthopanax sieboldianus*. *Nutrients* 5, 4134-4144. doi: 10.3390/nu5104134.

Kim, K.Y., Ku, S.K., Lee, K.W., Song, C.H., and An, W.G. (2018). Muscle-protective effects of Schisandrae Fructus extracts in old mice after chronic forced exercise. *J. Ethnopharmacol.* 212, 175-187. doi: 10.1016/j.jep.2017.10.022.

Kumar, R., Negi, P.S., Singh, B., Ilavazhagan, G., Bhargava, K., and Sethy, N.K. (2011). *Cordyceps sinensis* promotes exercise endurance capacity of rats by activating skeletal muscle metabolic regulators. *J Ethnopharmacol.* 136, 260-266. doi: 10.1016/j.jep.2011.04.040.

Lee, N., Lee, S.-H., Yoo, H.-R., Yoo, H. S. (2016). Anti-fatigue effects of enzyme-modified Ginseng extract: a randomized, double-blind, placebo-controlled trial. *J. Altern. Complem. Med.* 22, 859-864. doi: 10.1089/acm.2016.0057

Li, C.H. (2014a). Effect of Rhizoma Curculiginis on single contraction tension and mTOR activation of gastrocnemius muscle in rats. [master’s thesis]. [Qufu (SD)]: Qufu Normal University.

Li, D., Ren, J.W., Zhang, T., Liu, R., Wu, L., Du, Q., et al. (2018). Anti-fatigue effects of small-molecule oligopeptides isolated from *Panax quinquefolium* L. in mice. *Food Funct.* 9, 4266-4273. doi: 10.1039/c7fo01658a.

Li, G. (2018). Effects of ethanol extract of *Acanthopanax senticosus* seed on exercise fatigue of mice. *Chin. Oils Fats* 43**,** 75-77.

Li, L., Xiao, Y., Liu, Y., Gu, X., Yu, D., Li, W., et al. (2014). Main substance and biological activity of acidic components of *Schisandra chinensis*. *Chin. J. Exp. Trad. Med. Formula* 20, 70-73. doi: 10.11653/syfj2014050070.

Li, M. (2014b). Effects of *Polygonatum* polysaccharides on antioxidation and neurotransmitter in exercise fatigue rats. *Food Sci. Technol.* 39, 227-230. doi: 10.13684/j.cnki.spkj.2014.09.048.

Li, R. (2021). Purification of polysaccharide from Radix Paeoniae Alba and its activity of resistant exercise fatigue. *Chem. Eng.* 35, 69-73. doi: 10.16247/j.cnki.23-1171/tq.20210169.

Li, X.W. (2020b). Study on anti-fatigue effect and mechanism of *Polygonatum* polysaccharide*.* [master’s thesis]. [Hefei (AH)]: Hefei University of Technology.

Li, X.Y. (2020c). Effect of *Ganoderma lucidum* polysaccharide on exercise fatigue and exercise-induced immunosuppression. *Edible Fungi China* 39, 45-48. doi: 10.13629/j.cnki.53-1054.2020.02.013.

Li, Y. (2020a). The application of *Ophiocordyceps sinensis* to resist sports fatigue and enhance sports ability. *Edible Fungi China* 39**,** 35-37. doi: 10.13629/j.cnki.53-1054.2020.01.008.

Liang, X., Wei, B., Chen, Q., Ma, J., Li, K., and Huang, X. (2018). Optimization of polysaccharide extraction process from *Morinda officinalis* How and its biological activity in vitro and in vivo. *Food Mach.* 34, 158-163. doi: 10.13652/j.issn.1003-5788.2018.07.033.

Liang, Z., Chen, G., Long, S., Li, L., Li, L., Bai, J., et al. (2020). Anti-fatigue effect of hairy antler composition on mice and rats. *Chin. J. Comparative Med.* 30**,** 39-43. doi: 10. 3969 /j.issn.1671-7856. 2020. 03. 007.

Lin, M., Liu, Q., Liao, Y., Zheng, X., and Hu, X. (2019). Spectrum-effect relationship between serum fingerprints of Panacis Quinquefolii radix and its anti-fatigue effect. *Cent. South Pharm.* 17, 1014-1017. doi: 10.7539/j.issn.1672-2981.2019.07.008.

Liu, D., Shang, H., and Song, X. (2013). Antifatigue effect of extract from *Eucommia ulmoides* leaves purified by resin. *Food Sci.* 34**,** 251-254.

Liu, H.L., and Ling, L. (2012). The change in serum inflammatory cytokines level after exhaustive exercise based on administration of tanshinone ⅡA. *Chin. J. Sports Med.* 31, 999-1002+1020. doi: 10.16038/j.1000-6710.2012.11.011.

Liu, Y., Bao, X., Wang, J., Wei, C., and Bai, Y. (2021). Anti exercise fatigue and antioxidant of polysaccharide from *Hippophae rhamnoides*. *Sci. Technol. Food Ind.* 42, 321-326. doi: 10.13386/j.issn1002-0306.2020080192.

Liu, Y.P. (2012). Evaluation on *Saussurea involucrate* cell cultures for anti-oxidation & radioresistance and anti-fatigue*.* [master’s thesis]. [Dalian (LN)]: Dalian University of Technology.

Liu, Z., Nie, Y., and Liu, Q. (2017a). Regulation and control effects of Semen Astragali Complanati on GAD67, GABA-T and SSADH gene expression in the hippocampus of exercised rats. *Shandong Sports Sci. Tech.* 39, 53-58. doi: 10.14105/j.cnki.1009-9840.2017.01.012.

Liu, Z., Nie, Y., and Liu, Q. (2017b). Study on the regulatory effects of Semen Astragali Complanati on the amino acid neurotransmitters in the gyri hippocampi of exercise rats. *Chin. Sport Sci. Tech.* 53, 134-138. doi: 10.16470/j.csst.201702018.

Long, B., Xu, H.H., and Zhang, X.D. (2013). Anti-fatigue effect of *Morinda offieinalis* How. *Lishizhen Med. Mater. Med. Res.* 24, 298-300. doi: 10.3969/j.issn.1008-0805.2013.02.019.

Long, B.B. (2010). The effects of *Gynostemma* on sports ability of mice. *Chin. J. Appl. Phys.* 26, 339-340. doi: 10.13459/j.cnki.cjap.2010.03.010.

Luan, J., Chen, Y.L., Chu, Z.Y., and Wang, J.C. (2013). Effect of *Cordyceps sinensis* fruit body on anti-fatigue and anti-hypoxia abilities in mice. *Lishizhen Med. Mater. Med. Res.* 24, 47-48. doi: 10.3969/j.issn.1008-0805.2013.01.021.

Luo, X., Liu, W., Zhong, H., Yan, Y., Feng, F., and Zhao, M. (2021). Synergistic effect of combined oyster peptide and ginseng extracts on anti-exercise-fatigue and promotion of sexual interest activity in male ICR mice. *J. Funct. Foods* 86. doi: 10.1016/j.jff.2021.104700.

Ma, S., Chen, H., and Liang, Z. (2019). Intervention effect of cassia seed decoction combined with 4 weeks swimming training on exercise-induced fatigue in mice. *Chin. J. Appl. Phys.* 35, 522-524. doi: 10.12047 /j.cjap.5821.2019.114.

Ma,Y. (2019). Study on the effect of *Lycium barbarum* on testosterone concentration and anti-fatigue ability of exercise male rats. *Genomics and Applied Biology* 38, 5303-5307. doi: 10.13417/j.gab.038.005303.

Nie, D., TIAN, W., Jiang, B., Ma, X., Zhou, L., and Liu, X.M. (2014). Effects of *Phyllanthus emblica* on cerebral hypoxia and serum antioxidants after hypobaric exercise. *J. Chin. Med. Mater.* 37, 1448-1452. doi: 10.13863/j.issn1001-4454.2014.08.035.

Niu, Y. (2018). Effects of *Polygonatum odoratum* polysaccharide on anti-fatigue in mice. *Nat. Prod. Res. Dev.* 30, 1202-1207. doi: 10.16333/j.1001-6880.2018.7.018.

Qi, B., Liu, L., Zhang, H., Zhou, G.X., Wang, S., Duan, X.Z., et al. (2014). Anti-fatigue effects of proteins isolated from *Panax quinquefolium*. *J Ethnopharmacol.* 153, 430-434. doi: 10.1016/j.jep.2014.02.045.

Qi, S., and Xiong, Z. (2011). Effect of *Ligustrum lucidum* extract on antioxidation and exercise capacity of rat skeletal muscle. *J. Xi'an Jiaotong Univ. (Med. Sci.)* 32, 187-189+192.

Qi, S., and Xiong, Z. (2013). Effects of *Ligustrum lucidum* extract on brain tissue oxidative damage of rats taking high-intensity endurance training. *J. Northwest A. F. Univ. (Nat. Sci. Ed.)* 41, 30-34+40. doi: 10.13207/j.cnki.jnwafu.2013.02.034.

Qin, H., Li, S., Hu, Q., and Huan, F. (2019). Safety evaluation of freeze-dried powder of sika deer antler blood and its effect on alleviating physical fatigue in mice. *Food Mach.* 35, 161-166+176. doi: 10.13652/j.issn.1003-5788.2019.08.030.

Ren, M.J. (2020). Experimental study on the preventive effect of *Sedum aizoon* L. on exercise fatigue in mice and humans*.* [master’s thesis]. [Beijing (BJ)]: Capital University of Physical Education and Sports.

Shan L.N., and Shi Y.X. (2014). Effects of polysaccharides from *Gynostemma pentaphyllum* (Thunb.), Makino on physical fatigue. *Afr. J. Tradit. Complement Altern. Med.* 11, 112-117. doi: 10.4314/ajtcam.v11i3.17.

Shang, Y., and Li, J. (2018). Research on the mechanism of resistance to exercise-induced fatigue of *Codonopsis* extract. *J. Southwest Univ. (Nat. Sci. Ed.)* 40, 9-14. doi: 10.13718/j.cnki.xdzk.2018.06.002.

Shi, J., Zhao, Y., Lei, Y., and Zhang, R.J. (2011). Study on anti-fatigue and anti-oxidation effects of *Polygonatum* polysaccharide. *Lishizhen Med. Mater. Med. Res.* 22, 1409-1410. doi: 10.3969/j.issn.1008-0805,2011.06.058.

Song, J., Wang, Y., Teng, M., Cai, G., Xu, H., Guo, H., et al. (2015). Studies on the Antifatigue Activities of Cordyceps militaris Fruit Body Extract in Mouse Model. *Evid. Based Complement Alternat. Med.* 2015**,** 174616. doi: 10.1155/2015/174616.

Su, K.Y., Yu, C.Y., Chen, Y.W., Huang, Y.T., Chen, C.T., Wu, H.F., et al. (2014). Rutin, a flavonoid and principal component of *Saussurea involucrata*, attenuates physical fatigue in a forced swimming mouse model. *Int. J. Med. Sci.* 11, 528-537. doi: 10.7150/ijms.8220.

Su, X. (2020). Effects of different levels of *Hippophae rhamnoides* polysaccharide on sports performance and sugar metabolism of Mongolian horses. *Chin. Feed* 53-56. doi: 10.15906/j.cnki.cn11-2975/s.20201311.

Su, Y., and Qi, B. (2016). Protective effects of polysaccharides from *Gynostemma pentaphyllum* Makino on the exhaustive exercise-induced hepatocyte apoptosis. *Food Res. Dev.* 37, 158-162. doi: 10.3969/j.issn.1005-6521.2016.14.040.

Sumiyoshi, M., and Kimura, Y. (2016). Effects of *Eleutherococcus senticosus* Cortex on recovery from the forced swimming test and fatty acid β-oxidation in the liver and skeletal muscle of mice. *Nat. Prod. J.* 6, 49-55. doi: 10.2174/2210315506999151207145020.

Tan, W., Yu, K.Q., Liu, Y.Y., Ouyang, M.Z., Yan, M.H., Luo, R., et al. (2012). Anti-fatigue activity of polysaccharides extract from Radix Rehmanniae preparata. *Int. J. Biol. Macromol.* 50, 59-62. doi: 10.1016/j.ijbiomac.2011.09.019.

Tang, H.T. (2019). Improvement effect of sea horses on impaired memory induced by alcohol, depression induced by stress, and fatigue*.* [master’s thesis]. [Zhanjiang (GD)]: Guangdong Ocean University. doi: 10.27788/d.cnki.ggdhy.2019.000180.

Tao, Y., Jin, Q., and Xu, H. (2020). Effects of *Oyster* peptide supplementation and exercise training on exercise endurance in mice. *Food Sci. Technol.* 45, 57-63. doi: 10.13684/j.cnki.spkj.2020.03.014.

Wang, H., Shen, Z., Xiao, H., Liu, X., and Zhang, Y. (2019). Preliminary research on the anti-fatigue effect of *Ganoderma lucidum* spore powders and its biochemical mechanism. *Acta. Nutr. Sin.* 41, 173-177. doi: 10.13325/j.cnki.acta.nutr.sin.2019.02.014.

Wang, J. (2020). Effects of oyster polypeptide on mitochondrial function of skeletal muscle in exercise-induced fatigue rats. *J. Anhui Univ. Nat. Sci. (Nat. Sci. Ed.)* 44, 93-99. doi: 10. 3969/j. issn. 1000-2162. 2020. 05. 012.

Wang, X., Wang, Y., Wu, Z, and Xiong, Z. (2013). Experimental study of *Eucommia ulmoides* Oliver extract as anti-exercise-fatigue. *J. Northwest Univ. (Nat. Sci. Ed.)* 43, 64-69+74. doi: 10.16152/j.cnki.xdxbzr.2013.01.010.

Wang, X.J. (2012). Study on *Schisandra* polysaccharide capsule and its anti-fatigue health efficacy*.* [master’s thesis]. [Changchun (JL)]: Jilin Agricultural University.

Wang, Y., Liu, Q., and Geng, J. (2020). Purification and resisting movement fatigue activity of flavonoids from *Hippophae rhamnoides* L. *Sci. Technol. Food Ind.* 41, 169-174. doi: 10.13386/j.issn1002-0306.2020020153.

Wang, Y., Yu, X., Wu, X., Wang, H., Zhang, G., and Cui, P. (2014). Effects of *Polygonatum sibiricum* polysaccharides on free radical metabolism in brain tissue of mice at exhaustion and recovery. *Chin. J. Public Health* 30, 1165-1167. doi: 10.11847/zgggws2014-30-09-18.

Wang, Y., Zhou, W., Lyu, C., Li, Q., Kou, F., Jiang, M., et al. (2021). Metabolomics study on the intervention effect of radix Salviae miltiorrhizae extract in exercise-induced exhaustion rat using gas chromatography coupled to mass spectrometry. *J. Chromatogr. B.* 1178, 122805. doi: 10.1016/j.jchromb.2021.122805.

Wei, Y., Guo, Y., Li, M., Chen, L., Wang, Y., Jiang, S., et al. (2021). Anti-fatigue activity and mechanisms of peptides derived from *Perilla frutescens* seeds. *J. Chin. Inst. Food Sci. Technol.* 21, 157-162. doi: 10.16429/j.1009-7848.2021.07.019.

Wu, L., Ye, Q., and Qi, L. (2013). Effect of eleutheroside B on aerobic exercise-induced fatigue recovery. *Northwest Pharm. J.* 28, 50-53. doi: 10.3969/j.issn.1004-2407.2013.01.021.

Xia, S., and Pu, J. (2010). Study on extraction of polysaccharide from *Eucommia ulmoides* Oliver leaves and its anti-fatigue effect. *J. Anhui Agri. Sci.* 38, 18747-18748+18751. doi: 10.13989/j.cnki.0517-6611.2010.33.011.

Xing, X., and Ke, Y. (2019). Nutritional value of *Lycium ruthenicum* Murr. and its relieving resistance to exercise-induced fatigue. *Prog. Nutr.* 21, 876-881. doi: 10.23751/pn.v21i4.8524.

Xiong, B., Nie, Y., Wu, Z., Wang, X., and Xiong, Z. (2017). Study of complanatus child in Chinese herbal medicine as anti-fatigue action. *J. Northwest Univ. (Nat. Sci. Ed.)* 47, 87-91. doi: 10.16152/j.cnki.xdxbzr.2017-01-015.

Yan, W.J., Li, T.H., Lao, J.H., Shen, Y.H., and Fang, J.M. (2012). Anti-fatigue activity of hot aqueous extracts of *Ophiocordyceps sinensis* Mycelium evaluated using a mouse model. *Acta. Edulis. Fungi.* 19, 61-64. doi: 10.16488/j.cnki.1005-9873.2012.01.001.

Yang, J. (2013). Effects of Semen Astragali Complanati on 5-HT and relevant substances levels of brain in exercise-induced fatigue rats. *Chin. Sport Sci. Tech.* 49, 139-144. doi: 10.16470/j.csst.2013.04.022.

Yang, J., and Xiong, Z. (2010). Effects of Semen Astragali Complanati on BCAA and AAA levels of blood in exercise-induced fatigue rats. *J. Shaanxi Norm. Univ. (Nat. Sci. Ed.)* 38, 104-108. doi: 10.15983/j.cnki.jsnu.2010.02.019.

Yang, J., Wang, Q., Sun, J., and Feng, D. (2018a). Anti-fatigue and improvement effect of exhausting exercise-induced myocardial damage of aqueous extract from *Lycium ruthenicum* Murr. in mice. *Sci. Technol. Food Ind.* 39, 296-299+312. doi: 10.13386/j.issn1002-0306.2018.16.053.

Yang, X., Dai, P., Zeng, L., and Jiang, B. (2019). Anti-fatigue effect and mechanism of flavonoids from Rhizoma Polygonatum. *Modern Food* 134-137. doi: 10.16736/j.cnki.cn41-1434/ts.2019.08.038.

Yang, X., Geng, J., Qiu, D., Wang, X., and Luo, H. (2018b). Effects of *Lycium ruthenicum* Murr. aqueous extract on oxidative stress induced by exercise fatigue. *Chin. J. Mod. Appl. Pharm.* 35, 1494-1497. doi: 10.13748/j.cnki.issn1007-7693.2018.10.013.

Yeh, T.S., Huang, C.C., Chuang, H.L., and Hsu, M.C. (2014). *Angelica sinensis* improves exercise performance and protects against physical fatigue in trained mice. *Molecules* 19, 3926-3939. doi: 10.3390/molecules19043926.

Yin, A., Tian, R., and Wang, P. (2012). Study on anti-fatigue effect of *Semen Cuscutae* crude extracts. *Sci. Technol. Food Ind.* 33, 164-165+182. doi: 10.13386/j.issn1002-0306.2012.14.014.

Zhang, G., Zhou, S., Tian, H., Huang, Q., and Gao, Y. (2011c). An experimental study of *Phyllanthus emblica* L. on the anti-fatigue effect on mice in simulated plateau environment. *Pharm. J. Chin. PLA.* 27, 208-211. doi: 10.3969/j.issn.1008-9926.2011.03.07.

Zhang, Q., Feng, Q., Zhou, Z., Li, R., Fang, L., Yang, Q., et al. (2020a). Improvement effect of eleutheroside B on learning and memory abilities of fatigue mice and its mechanism of activating Keap1/Nrf2/ARE signaling pathway. *J. Jilin Univ. (Med. Ed.)* 46, 771-778. doi: 10.13481/j.1671-587x.20200417.

Zhang, R., Zhao, Y., and Wang, Z. (2011a). Anti-fatigue effects of antler velvet water extract in mice. *Sci. Technol. Food Ind.* 32, 365-367. doi: 10.13386/j.issn1002-0306.2011.04.016.

Zhang, S., Wang, M., Cheng, X., Zhang, Qi., and Wu, P. (2021). Ultrahigh pressure extraction of *Polygonatum sibiricum* polysaccharide and mechanism of improving exercise endurance. *J. Nucl. Agric. Sci.* 35, 2094-2101. doi: 10.11869/j.issn.100-8551.2021.09.2094.

Zhang, S.S. (2021). The effect and mechanisms of cordycepin on anti exercise-induced fatigue in mice. [master’s thesis]. [Nanchang (JX)]: Jiangxi Science and Technology Normal University.

Zhang, W., Yu, X., Wang, Z., and Lin, R. (2012). Observe the role of different doses of tanshinone Ⅱ_A_ sodium for heart muscular tissue of rats after exhaustive exercise. *Chin Med Pharm* 2**,** 32-34.

Zhang, X., Lin, H., Li, X., Jing, S., Sun, W., Jiang, W., et al. (2020b). Schisantherin A improves fatigue in mice by regulating the Nrf2/ARE antioxidant pathway in liver. *Food Sci.* 41, 190-195. doi: 10.7506/spkx1002-6630-20181120-230.

Zhang, X.L., Ren, F., Huang, W., Ding, R.T., Zhou, Q.S., and Liu, X.W. (2010). Anti-fatigue activity of extracts of stem bark from *Acanthopanax senticosus*. *Molecules* 16, 28-37. doi: 10.3390/molecules16010028.

Zhang, X.W. (2010). The composition and anti-fatigue activity of the lipid-soluble fraction of velvet antler. [master’s thesis]. [Chongqing (CQ)]: southwest university.

Zhang, Y., Zhang, L., Li, T., Peng, J., and Cui, M. (2011b). Study on the anti-inflammatory effect, analgesic, anti-fatigue and hypoxia tolerance of Taishan *Hippophae rhamnoides* fruits. *Sci. Technol. Food Ind.* 32**,** 377-378+381. doi: 10.13386/j.issn1002-0306.2011.03.025.

Zhang, Z., and Xu, S. (2011). Effects of *Schisandra chinensis* polysaccharides on anti-fatigue and anti-hypoxia in mice. *J. Northeast Forest. Univ. (Chin. Ed.)* 39, 98-99+103. doi: 10.13759/j.cnki.dlxb.2011.12.019.

Zhao, K. (2019). *Cordyceps sinensis* active extract cordyceps polysaccharide combined with aerobic exercise improvement suggestions. *Edible Fungi China* 38, 42-45. doi: 10.13629/j.cnki.53-1054.2019.02.012.

Zhao, Z., Zheng, X., and Fang, F. (2014). *Ganoderma lucidum* polysaccharides supplementation attenuates exercise-induced oxidative stress in skeletal muscle of mice. *Saudi. J. Biol. Sci.* 21, 119-123. doi: 10.1016/j.sjbs.2013.04.004.

Zhong, L., Zhao, L., Yang, F., Yang, W., Sun, Y., Hu, Q. (2017). Evaluation of anti-fatigue property of the extruded product of cereal grains mixed with *Cordyceps militaris* on mice. *J. Int. Soc. Sports Nutr*. 14, 15. doi: 10.1186/s12970-017-0171-1.

Zhou, S., Tian, H., Huang, Q., Gao, Y., and Zhang, G. (2013). Anti-fatigue function of *Panax quinquefoiuml* in mice under simulated plateau conditions. *Pharm. J. Chin. PLA.* 29, 297-300. doi: 10.3969/j.issn.1008-9926.2013.04.006.

Zhu, C., Cao, J., Zhou, H., Cheng, Y., and Shang, X. (2013). Effect of Morindae Officinalis Radix on the exercise capacity in rats and oxidation resistance of mitochondrial. *Chin. J. Exp. Trad. Med. Formula* 19, 219-222. doi: 10.13422/j.cnki.syfjx.2013.03.072.

Zhu, G. (2010). Effect of Semen Astragali Complanati on exercise training rats’ sugar metabolism and contents of serum urea nitrogen and blood lactate. *J. Northwest Univ. (Nat. Sci. Ed.)* 40, 655-658. doi: 10.16152/j.cnki.xdxbzr.2010.04.038.
